# Development of MTH1-Binding Nucleotide Analogs Based on 7,8-Dihalogenated 7-Deaza-dG Derivatives

**DOI:** 10.3390/ijms22031274

**Published:** 2021-01-28

**Authors:** Hui Shi, Ren Ishikawa, Choon Han Heh, Shigeki Sasaki, Yosuke Taniguchi

**Affiliations:** 1 Graduate School of Pharmaceutical Sciences, Kyushu University, 3-1-1 Maidashi, Higashi-ku, Fukuoka 812-8582, Japan; shihui19910716@yahoo.co.jp (H.S.); glaylarc.yfm1003@gmail.com (R.I.); sigesasaki@niu.ac.jp (S.S.); 2 Department of Pharmacy, Faculty of Medicine, University of Malaya, Kuala Lumpur 50603, Malaysia; silverbot@um.edu.my; 3 Graduate School of Pharmaceutical Sciences, Nagasaki International University, 2825-7 Huis Ten Bosch Machi, Sasebo City, Nagasaki 859-3298, Japan

**Keywords:** oxidized nucleotide, MTH1, dihalogenated nucleoside derivative, nucleotide analog

## Abstract

MTH1 is an enzyme that hydrolyzes 8-oxo-dGTP, which is an oxidatively damaged nucleobase, into 8-oxo-dGMP in nucleotide pools to prevent its mis-incorporation into genomic DNA. Selective and potent MTH1-binding molecules have potential as biological tools and drug candidates. We recently developed 8-halogenated 7-deaza-dGTP as an 8-oxo-dGTP mimic and found that it was not hydrolyzed, but inhibited enzyme activity. To further increase MTH1 binding, we herein designed and synthesized 7,8-dihalogenated 7-deaza-dG derivatives. We successfully synthesized multiple derivatives, including substituted nucleosides and nucleotides, using 7-deaza-dG as a starting material. Evaluations of the inhibition of MTH1 activity revealed the strong inhibitory effects on enzyme activity of the 7,8-dihalogenated 7-deaza-dG derivatives, particularly 7,8-dibromo 7-daza-dGTP. Based on the results obtained on kinetic parameters and from computational docking simulating studies, these nucleotide analogs interacted with the active site of MTH1 and competitively inhibited the substrate 8-oxodGTP. Therefore, novel properties of repair enzymes in cells may be elucidated using new compounds.

## 1. Introduction

Reactive oxygen species (ROS) are a product or byproduct of cellular metabolism; some are generated in the mitochondria of cells, while others are exogenously produced, such as air and water pollution, cigarette smoke, alcohol, heavy or transition metals, and certain drugs [1,2]. ROS include oxygen-derived radicals and non-radical species, such as the hydroxyl radical, superoxide ion, hydrogen peroxide, and peroxynitrite. These ROS are involved in several signaling pathways and affect cellular function. However, they also have the potential to induce damage to lipids, proteins, and DNA [3]. Free nucleotides are particularly vulnerable to oxidative damage by ROS. The major forms of oxidative damage induced by ROS in the nucleotide pool are 8-oxo-dGTP and 2-OH-dATP [4,5,6,7]. 8-oxo-dGTP prefers the *syn*-conformation at glycosidic bonds and is incorporated into the duplex DNA strand not only for the dC-template, but also dA-template at the stage of DNA replication steps [8,9,10,11]. This property of 8-oxo-dGTP for the dA-template leads to a genetic mutation known as the TA to GC transversion mutation [12,13,14]. The nucleotide repair enzyme, MTH1 (human mutt homolog 1, also called NUDT1), may hydrolyze 8-oxo-dGTP to its monophosphate, 8-oxo-dGMP, to prevent its mis-incorporation into duplex DNA during DNA replication steps [15,16,17].

In 2014, Gad and Huber et al. reported that the inhibition of MTH1 function by the RNAi-mediated knockdown of expression or by novel compounds (including TH588, TH287, and Crizotinib) exerted strong anti-proliferation effects in various cancer cells, but not normal cells [18,19,20]. The inhibition of MTH1 has been proposed to increase the incorporation of 8-oxo-dGTP in cells, thereby enhancing DNA damage and, ultimately, resulting in cancer cell death [21,22,23]. In contrast, recent studies suggested that MTH1 does not affect the survival of cancer cells [24,25,26].

**Figure 1:**
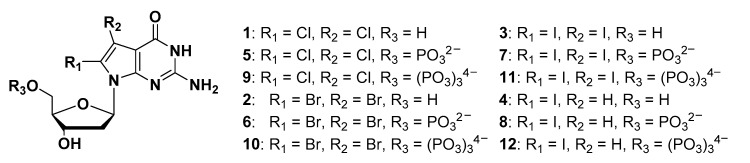
Structures of 8-substituted 7-deaza-dG derivatives and 7,8-disubstituted 7-deaza-dG derivatives.

To address this discrepancy and reveal its functions or properties in living cells, we developed 8-halogenated 7-deaza-dGTP, an 8-oxo-dGTP analogue [27,28,29], and γ-*N*-modified 8-oxo-dGTP [30] as a MTH1-binding compound. This structural mimicry of a natural product is important for elucidating the functions or mechanisms of intracellular events. We demonstrated that 8-iodo-7-deaza-dGTP inhibited the hydrolytic activity of MTH1, the IC_50_ value of which was 0.42 μM and *K*_i_ value was 61.7 nM. This was the first example of the development of a MHT1 inhibitor using nucleotide derivatives, which prompted us to conduct further experiments. Based on the predicted complex structure between 8-iodo-7-deaza-dGTP and MTH1, a space exists between the active site of MTH1 and the 8-position of the nucleobase. Therefore, in the present study, we designed 7,8-dihalogenated 7-deaza-dG derivatives as novel MTH1-binding compounds to increase binding affinity (Figure 1).

## 2. Results and Discussion

### 2.1. Synthesis of New 7-Deaza-dG Derivatives

**Scheme 1:**
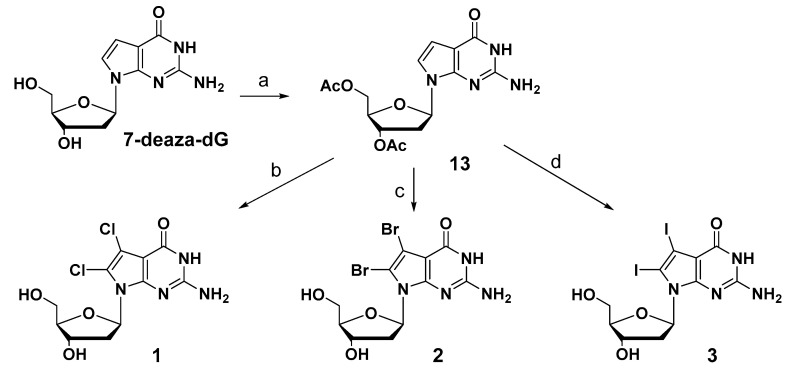
Reagents and conditions: (**a**) acetic anhydride, anhydrous pyridine, r.t. 91%; (**b**) (1) *N*-chlorosuccinimide (NCS) (2.0 eq), anhydrous DMF, r.t., 1 h; (2) 7N ammonium in methanol, r.t., 18 h, 36% for two steps; (**c**) (1) NBS (2.0 eq), anhydrous DMF, r.t., 1 h; (2) 7N ammonium in methanol, r.t., 18 h, 35% for two steps. (**d**) (1) *N*-iodosuccinimide (NIS) (2.5 eq), anhydrous DMF, r.t., 1 h; (2) 7N ammonium in methanol, r.t., 18 h, 18% for two steps.

7-Deaza-2′-deoxyguanosine (7-deaza-dG) was used as the starting material to synthesize newly designed substituted derivatives. The synthetic schemes of these derivatives are summarized in Scheme 1. The 3′- and 5′-hydroxyl groups of 7-deaza-dG were protected with an acetyl group by using acetic anhydride in pyridine. The acetylated compound (**13**) was then treated with the 2.0 equivalent of *N*-chlorosuccinimide (NCS) to modify the 7- and 8-positions of 7-deazaguanine using a chlorination reaction. In contrast to the previously reported regioselective reaction at the 8-position, a dihalogen-introduced compound was obtained at the 7- and 8-positions under this condition in 49% yield. Although the solvent, reaction time, and temperature were changed to improve yield, satisfactory yield was not obtained. 7,8-Dibromo-7-deaza-dG and 7,8-diiodo-7-deaza-dG were also obtained using a 2.0 equivalent of *N*-bromosuccinimide (NBS) and 2.5 equivalent of *N*-iodosuccinimide (NIS) in 51% and 51% yield, respectively. After removing the acetyl groups at the 5′- and 3′-hydroxyl groups, the corresponding 7,8-dihalogenated 7-deaza-dGs (**1**, **2**, and **3**) were obtained.

**Scheme 2:**
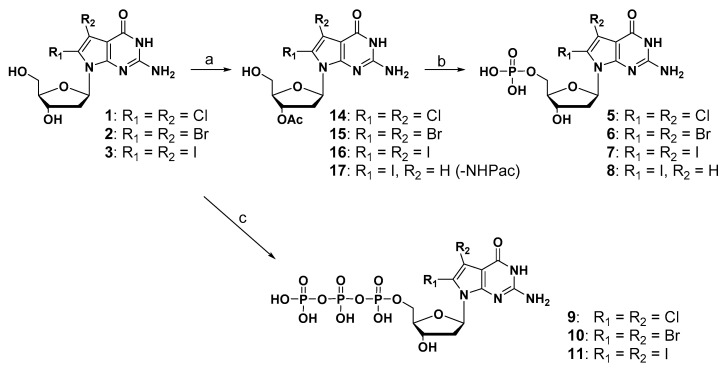
Reagents and conditions: (**a**) (1) TBSCl, dry pyridine, imidazole, DMAP, r.t., overnight, 37–71%; (2) acetic anhydride, dry pyridine, r.t., overnight, 49–92%; (3) Et_3_N-3HF, TEA, dry THF, r.t., 5 h, 91–98%; (**b**) (1) barium-2-cyanoethylphosphate hydrate, dowex (50) H^+^, DCC; (2) 28% ammonia solution, r.t., 48 h, 9–31%; (**c**) (1). 2-Chloro-4*H*-1,3,2-benzodioxa-phosphorin-4-one, tributyl-ammonium pyrophosphate, tributylamine, dry DMF; (2). I_2_ in pyridine/H_2_O = 98:2 solution; (3) 28% ammonia solution, r.t., 14–30%.

The synthesis of monophosphate and triphosphate derivatives is depicted in Scheme 2. Regarding the synthesis of a monophosphate compound, when treated with POCl_3_, which is a general method, the dehalogenation reaction proceeded and the desired product was not obtained. Therefore, to prevent the decomposition of halogen substituents under strong acidic conditions [31], the diol compounds were converted into the corresponding 3’-acetylated compounds (**14**, **15**, and **16**) in three steps. These 3’-acetylated compounds (**14**, **15**, and **16**) and 2-*N*-phenoxylacetyl-3′-*O*-acetyl-8-iodo-7-deaza-dG (**17**) were treated with 2-cyanoethylbarium phosphate hydrate under mild phosphorylation reaction conditions, followed by ammonia solution to give the corresponding monophosphate derivatives (**5**, **6**, **7**, and **8**). The synthesis of triphosphate derivatives was performed in a one-step reaction [32,33]. 2-Chloro-4-*H*-1,3,2-benzodioxaphosphoryl-4-one and pyrophosphate were used to prepare a bulky phosphorylation reagent, which selectively phosphorylates at the 5′-hydroxyl group of the diol compound under mild conditions. After the oxidation of phosphite and the hydrolysis of the cyclic structure, the corresponding triphosphate derivatives (**9**, **10**, and **11**) were successfully synthesized in moderate yield.

### 2.2. Binding Properties of Dihalogenated-deaza-dG Derivatives for MTH1

#### 2.2.1. Hydrolytic Properties of New 7-Deaza-dGTP Derivatives by MTH1

**Figure 2:**
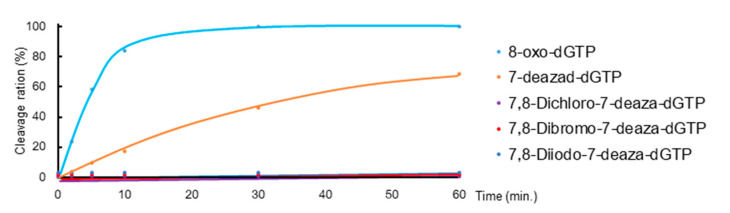
Time courses of the hydrolysis of 8-oxo-dGTP, 7-deaza-dGTP, and 7,8-dihalogenated-7-deaza-dGTP (**9**, **10**, and **11**) by MTH1. Reaction conditions: 20 mM Tris-HCl pH 7.5, 4 mM MgCl_2_, 40 mM NaCl, 80 μg/mL bovine serum albumin (BSA), 8 mM dithiothreitol (DTT), 10% glycerol; 50 μM 8-oxo-dGTP, 5 nM MTH1; 50 μM dNTPs; incubated at 37 °C for various times. Hydrolysis reactions were analyzed by HPLC, and the cleavage rate was calculated from the peak area.

Since the shapes of synthesized triphosphate derivatives were similar to 8-oxo-dGTP, we investigated whether these triphosphate derivatives are hydrolyzed by MTH1. MTH1 and these triphosphate derivatives were mixed and incubated at 37 °C for various times, and hydrolysis reactions were analyzed by HPLC. Hydrolyzed ratios were calculated from the peak area, which was summarized in Figure 2 as a time course plot. 8-Oxo-dGTP was rapidly hydrolyzed to its monophosphate, 8-oxo-dGMP, by MTH1 (Figure 2. sky-blue line). The triphosphate group of 7-deaza-dGTP was also hydrolyzed, but more slowly than 8-oxo-dGTP (Figure 2. orange line). However, in the case of 7,8-dihalogenated triphosphate derivatives, the peak of the corresponding monophosphate was not detected in HPLC following the treatment with MTH1 (Figure 2. **9**: purple, **10**: red and **11**: blue lines). Although these results were similar to those obtained for 8-iodo-7-deaza-dGTP, they indicated that these triphosphates were poor substrates or had no binding property for the active site of MTH1.

#### 2.2.2. Inhibitory Effects of New 7-Deaza-dG Derivatives on the Hydrolytic Activity of MTH1

We examined whether the nucleoside diols, nucleoside monophosphate (NMP), and nucleoside triphosphate (NTP) derivatives inhibit the MTH1 hydrolysis of 8-oxo-dGTP. MTH1 and 8-oxo-dGTP were mixed with various concentrations of synthesized derivatives, and this mixture was incubated and analyzed by HPLC. The inhibition efficiencies of IC_50_ values were calculated and summarized in Table 1. In the case of diol compounds, 7,8-dichloro-7-deaza-dG (**1**), 7,8-dibromo-7-deaza-dG (**2**), and 7,8-diiodo-7-deaza-dG (**3**) exerted inhibitory effects on the hydrolytic activity of MHT1 at a micromolar level (Table 1, Entries 1–3). Their IC_50_ values were better than that of 8-iodo-7-deaza-dG (**4**) (Table 1, Entry 4).

We then examined monophosphate compounds, the structures of which were products of a hydrolyzed reaction. The IC_50_ values of these compounds were markedly smaller than that of the diol compound (IC_50_ values: **5** is 0.13 μM, **6** is 0.26 μM, **7** is 0.13 μM, and **8** is 0.84 μM in Table 1, Entries 5–8.). These IC_50_ values were approximately 100-fold smaller for each corresponding diol compound. These results indicated that the monophosphate moiety strongly interacted with the enzyme, and this interaction was also observed in 8-oxo-dGMP. We then assessed the inhibitory effects of triphosphate derivatives (Entries 9–12) on MTH1 activity. The IC_50_ values of dihalogenated triphosphate analogs were not better than those of the corresponding monophosphate compounds (Entries 9–11 vs. 5–7); however, in many cases of triphosphate modifications, the value was markedly better than those of monophosphates. These results indicated that the phosphate group at the α-position was very important for the interaction with the enzyme, and modifying not only the 8-position, but also the 7-position with a halogen atom further contributed to binding to the enzyme and the inhibition of enzyme activity. Since the influence of the size of the halogen atom was small, these effects were attributed more to the electronic change of the nucleobase position, i.e., stacking interaction, than to the direct interaction of the halogen atom with the enzyme. Compared with the IC_50_ value of TH287 (Table 1, Entry 13) [28], which is the strongest class inhibitor as a small molecule, the IC_50_ value of our triphosphate compounds were about 10-fold different. These results were also shown to be very interesting as an inhibitory effect on MTH1 activity.

#### 2.2.3. Assessment of Inhibition Modes of New 7-Deaza-dG Derivatives

We made a Lineweaver-Burk plot to calculate kinetic parameters (*K*_M_ and *k*_cat_) in order to reveal the types of inhibition modes for MTH1. Kinetic parameters were summarized in Table 2. In the absence of inhibitors, *k*_cat_ was 10.6 s^−1^, and *K*_M_ was 17.6 μM (Entry 1). In the presence of new 7-deaza-dG monophosphate and triphosphate, *k*_cat_ values were only slightly altered (Entries 2–7). On the other hand, all *K*_M_ values increased (Entries 2–7). These results clearly demonstrated that the inhibition modes of new compounds involve competitive inhibition with the substrate of 8-oxo-dGTP. Among these derivatives, 7,8-dibromo-7-deaza-dGTP (**10**) showed the best *K*_i_ value (24.2 nM) in this experiment (Entry 5).

#### 2.2.4. Prediction of Binding Properties of New 7-Deaza-dG Derivatives with MTH1

**Figure 3:**
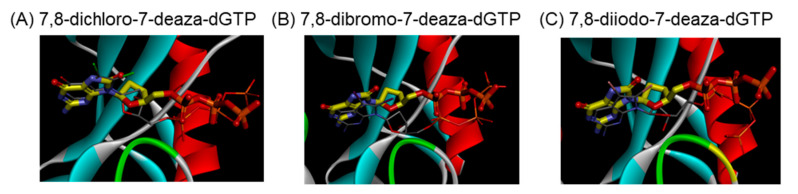
Images of optimal docking conformations of (**A**) 7,8-dichloro-7-deaza-dGTP (**7**) (thin stick with nitrogen colored in blue, oxygen colored in red, phosphorus colored in orange and chlorine colored in green), (**B**) 7,8-dibromo-7-deaza-dGTP (**10**) (thin stick with bromine colored in reddish brown), and (**C**) 7,8-diiodo-7-deazadGTP (**13**) (thin stick with iodine colored in light purple) in the binding pocket of MTH1 relative to 8-oxo-dGTP (thick stick with carbon colored in yellow).

**Figure 4:**
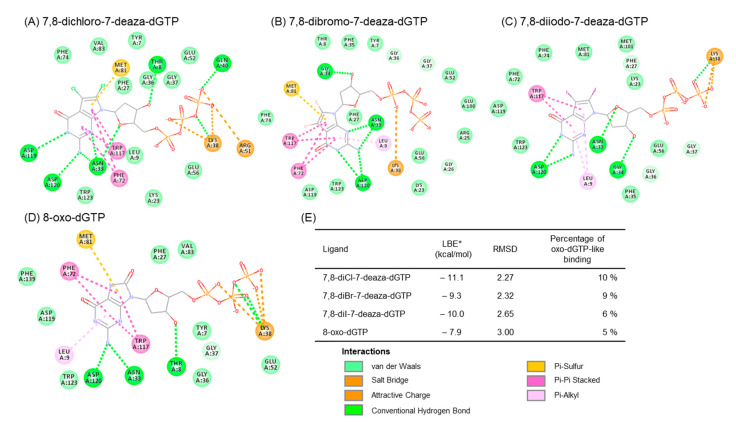
2D-Interaction diagram between docked MTH1 and (**A**) 8-oxo-dGTP (PDB: 5FSI), (**B**) 7,8-dichloro-7-deaza-dGTP (**7**), (**C**) 7,8-dibromo-7-deaza-dGTP (**10**), (**D**) 7,8-diI-7-deazadGTP (**13**), and (**E**) and binding properties. * LBE (Lowest binding energy) value for the conformation with RMSD < 5 Å compared to the 8-oxo-dGTP moiety in the crystal structure 5FSI. Percentage of conformations with the ability to bind to the active site of hMTH1 with RMSD < 5 Å. One hundred conformations (n-100) will be generated as the docking resulting.

We predicted the complex structure between 8-dihalogenated-7-deaza-dGTP (**7**, **10**, and **13**) and MTH1 using a docking study. The best docking conformations were shown in Figure 3 and the corresponding 2D-interaction diagrams were depicted in Figure 4. The docking results showed that the positions of the nucleobase part were similar to the substrate, 8-oxo-dGTP. Since these derivatives, such as 8-oxo-dGTP, have the ability to form a hydrogen bond at the Watson-Click phase of them (Figure 3, thin stick vs. thick stick structures). On the other hand, their sugar and triphosphate parts slightly shifted from those of 8-oxo-dGTP in the MTH1 active site. Although the underlying mechanisms currently remain unknown, this shift may be one reason why these 7,8-dihalogenated-7-deaza-dGTP derivatives were not hydrolyzed by MTH1. In addition, the binding characteristics of these 7,8-dihalogenated-7-deaza-dGTP derivatives to MTH1 revealed that their nucleobases and phosphate groups mainly and consistently interacted with Asp120, Trp117, and Lys38, respectively, with these amino acid showing identical interactions to those in 8-oxo-dGTP (Figure 4A–D). However, these 7,8-dihalogenated-7-deaza-dGTP derivatives showed better binding profiles in terms of binding energy and percentage binding than 8-oxo-dGTP (Figure 4E). Therefore, the introduction of a halogen atom into the nucleobase portion may increase binding due to changes in the surrounding electric environment, which may further enhance the pi-pi stacking interaction with the amino acid residue, Trp117 at the active site of MTH1.

Although these compounds that showed very interesting binding properties, these triphosphates are expected to be very high water solubility and polarity. These are also supported by the ADME prediction results (Supplementary Figures S1–S4). Since the γ-position of triphosphate group placed the outside of binding pocket of the enzyme from the docking study, the further chemical modifications of this part are possible.

## 3. Materials and Methods

### 3.1. Synthesis of New 7-Deaza-dG Derivatives

General: ^1^H NMR, ^13^C NMR, and ^31^P-NMR spectra was measured with 500 MHz (Ascend 500, Bruker, Billerica, MA, USA), 400 MHz (UNITY 400, Agilent, Santa Clara, CA, USA) nuclear magnetic resonance instruments. Merck TLC Plate Silica Gel 60 F254 was used to detect reactions and column chromatography was conducted with silica gel 60N (Kanto Chemical Co., Inc., Tokyo, Japan). YAMAZEN AI-580S (Yamazen Co., Osaka, Japan) was used for flash column chromatography. MALDI-TOF mass spectra were recorded in the negative mode by Bruker Daltonics Microflex. HPLC purification (JASCO Co., Tokyo, Japan) and analyses were performed with reverse-phase C18 columns, including SHISEIDO CAPCELL PAK C18 MG (4.6 × 250 mm) and COSMOSIL 5C18-AR-II (4.6 × 250 mm) (Nacalai Tesque, Kyoto, Japan).

#### 3.1.1. General Procedure for the Synthesis of 7,8-Dihalogeneted-7-deaza-2′-deoxy-guanosine

3′,5′-di-*O*-Acetyl-7-deaza-2′-deoxyguanosine (**13**) (1 g, 2.86 mmol) was dissolved in 48 mL anhydrous DMF under an argon atmosphere at room temperature. After covering the flask, the 2 equivalent of NCS or NBS or 2.5 equivalent of NIS was added in two portions over one hour. The organic solvent was concentrated, and the resulting residue was purified by column chromatography (silica gel, CHCl_3_/MeOH = 30/1) to give a white solid. This solid was suspended in 20 mL of 7 N ammonium in methanol solution. The mixture was stirred at room temperature overnight under argon, concentrated, and the residue was purified by column chromatography (silica gel, CHCl_3_/MeOH = 10/1) to give a white solid.

7,8-di-Chloro-7-deaza-2′-deoxy-guanosine (**1**): Yield was 36% for two steps. ^1^H-NMR (500 MHz, CD_3_OD) δ (ppm): 6.41 (t, 1H, *J* = 1.8 Hz), 4.57–4.55 (m, 1H), 3.99–3.95 (m, 1H), 3.95–3.81 (m, 1H), 3.76–3.72 (m, 1H), 3.14–3.08 (m, 1H), 2.19–2.14 (m, 1H). HR-ESI-MS (*m*/*z*) calcd. for C_11_H_12_N_4_O_4_Cl_2_Na [M + Na]^+^: 357.0128, 359.0099, 361.0089, Found: 357.0159, 359.0138, 361.0130.

7,8-di-Bromo-7-deaza-2′-deoxy-guanosine (**2**): Yield was 35% for two steps. ^1^H-NMR (500 MHz, CD_3_OD) δ (ppm): 6.45–6.42 (m, 1H), 4.59–4.56 (m, 1H), 3.98–3.96 (m, 1H), 3.86–3.82 (m, 1H), 3.77–3.74 (m, 1H), 3.17–3.11 (m, 1H), 2.17–2.13 (m, 1H). HR-ESI-MS (*m*/*z*) calcd. for C_11_H_12_N_4_O_4_Br_2_Na [M + Na]^+^: 444.9118, 446.9097, 448.9077, Found: 444.9112, 446.9078, 448.9067.

7,8-di-Iodo-7-deaza-2′-deoxy-guanosine (**3**): Yield was 18% for two steps. ^1^H-NMR (500 MHz, DMSO-*d6*) δ (ppm): 10.62 (bs, 1H), 6.29 (t, 1H, *J* = 2.0 Hz), 6.24 (bs, 2H), 5.19 (d, 1H, *J* = 4.5 Hz), 4.94–4.92 (m, 1H), 4.39–4.36 (m, 1H), 3.76–3.75 (m, 1H), 3.65–3.64 (m, 1H), 3.57–3.52 (m, 1H), 3.16–3.08 (m, 1H), 2.01–1.98 (m, 1H). HR-ESI-MS (*m*/*z*) calcd. for C_11_H_13_N_4_O_4_I_2_Na [M + Na]^+^: 540.8841, Found: 540.8844.

#### 3.1.2. General Procedure for the Synthesis of 3′-*O*-Acetylated Compounds

The corresponding diol compounds (**1**, **2**, and **3**) (0.24 mmol) were dissolved in anhydrous pyridine (1.2 mL) under an argon atmosphere at room temperature, and *tert*-butyldimethylsilyl chloride (0.34 mmol), imidazole (0.48 mmol), and dimethylaminopyridine (DMAP) (0.05 mmol) were then added to the reaction mixture. The resulting mixture was stirred at room temperature overnight. The reaction mixture was concentrated under reduced pressure, and the residue was purified by flash silica-gel chromatography (Flash column, 10 g, CHCl_3_/MeOH = 100/0 to 86/14) to give a 5′-OTBS protected compound as a white powder. Acetic anhydride (0.95 mmol) was dropped into the solution of the above compound (0.24 mmol) in anhydrous pyridine (2.4 mL), and the reaction mixture was stirred overnight. The solvent was concentrated under reduced pressure, and the residue was purified by flash silica-gel chromatography (Flash column, 10 g, CHCl_3_/MeOH = 100/0 to 86/14) to give 3′-OAc and 5′-OTBS-protected compounds as a white powder. This compound (0.24 mmol) was dissolved in dry THF (2 mL). Triethylamine trihydrofluoride (0.33 mmol) and triethylamine (0.19 mmol) were then dropped into the reaction mixture at room temperature under an argon atmosphere. The reaction solution was stirred at room temperature for 5 h. After the removal of the solvent under reduced pressure, the residue was purified by flash silica-gel chromatography (Flash column, 10 g, CHCl_3_/MeOH = 100/0 to 95/5) to give **14**, **15**, and **16** as a white powder.

3′-*O*-Acetyl-7,8-di-chloro-7-deaza-2′-deoxy-guanosine (**14**): Yield was 40% for three steps. ^1^H-NMR (500 MH_Z_, DMSO-*d6*) δ (ppm): 10.98 (bs, 1H), 6.46 (bs, 2H), 6.36–6.29 (m, 1H), 5.31–5.29 (m, 1H), 4.92 (t, 1H, *J* = 6.4 Hz), 3.96–3.93 (m, 1H), 3.66–3.62 (m, 1H), 3.60–3.54 (m, 1H), 3.27–3.23 (m, 1H), 2.28–2.24 (m, 1H), 2.07 (s, 3H). HR-ESI-MS (*m*/*z*) calcd. for C_13_H_14_N_4_O_5_Cl_2_Na [M + Na]^+^: 399.0234, 401.0204, 403.0175, Found: 399.0252, 401.0225, 403.0199.

3′-*O*-Acetyl-7,8-di-bromo-7-deaza-2′-deoxy-guanosine (**15**): Yield was 59% for three steps. ^1^H-NMR (500 MH_Z_, DMSO-*d6*) δ (ppm): 10.75 (bs, 1H), 6.40 (bs, 2H), 6.30–6.27 (m, 1H), 5.33–5.31 (m, 1H), 4.94 (t, 1H, *J* = 6.3 Hz), 3.97–3.96 (m, 1H), 3.68–3.66 (m, 1H), 3.61–3.57 (m, 1H), 3.38–3.36 (m, 1H), 2.27–2.23 (m, 1H), 2.07 (s, 3H). HR-ESI-MS (*m*/*z*) calcd. for C_13_H_15_N_4_O_5_Br_2_ [M + H]^+^: 464.9404, 466.9383, 468.9363, Found: 464.9437, 466.9420, 468.9398.

3′-*O*-Acetyl-7,8-di-iodo-7-deaza-2′-deoxy-guanosine (**16**): Yield was 18% for three steps. 1H-NMR (500MHZ, DMSO) δ (ppm): 10.67 (s, 1H), 6.29–6.27 (m, 3H), 5.35–5.32 (m, 1H), 5.02 (t, 1H, *J* = 6.3 Hz), 3.98–3.96 (m, 1H), 3.72–3.67(m, 1H), 3.63–3.58 (m, 1H), 3.44–3.38 (m, 1H), 2.23–2.19 (m, 1H), 2.07 (s, 3H). HR-ESI-MS (*m*/*z*) calcd. for C_13_H_14_N_4_O_5_Cl_2_Na [M + Na]^+^: 582.8946, found: 582.8971.

#### 3.1.3. General Procedure for the Synthesis of 5′-Monophosphate Compounds

The corresponding 3′-OAc compounds (**14**, **15**, **16**, and **17** [28]) (0.021 mmol) and 2-cyanoethylphosphate (0.11 mmol) were dissolved in pyridine (0.58 mL), under an argon atmosphere at room temperature, and DCC (0.15 mmol) were then added to the reaction mixture. The resulting mixture was stirred at room temperature for 48 h. The reaction mixture was concentrated under reduced pressure, and then the residue was dissolved in 28% ammonia solution and stirred for 6 h at 40 °C. The reaction mixture was filtered off, and the filtrate was washed with diethyl ether, then aqueous layer was concentrated under reduced pressure. The resulting residue was purified by reversed-phase HPLC to give corresponding monophosphate compounds as a white material after lyophilization. (HPLC conditions: A: 0.1 M TEAA, B: MeCN; Flow rate: 1.0 mL/min; Shiseido MG1 (4.6 × 250 mm); UV detector: 254 nm; 35 °C).

7,8-di-Chloro-7-deaza-2′-deoxy-guanosine monophosphate (**5**): Yield was 31% for two steps. ^1^H-NMR (500 MH_Z_, D_2_O) δ (ppm) 6.48 (t, 1H, *J* = 9.6 Hz), 4.87–4.84 (m, 1H), 4.09–4.01 (m, 2H), 3.96–3.92 (m, 1H), 3.26–3.21 (m, 1H), 2.37–2.32 (m, 1H). ^31^P-NMR (202 MHz, D_2_O) δ (ppm) 3.79. HR-ESI-MS (*m*/*z*) calcd. for C_11_H_12_N_4_O_7_Cl_2_P [M − H]^−^: 412.9826, 414.9797, 416.9767, found: 412.9826, 414.9805, 416.9781.

7,8-di-Bromo-7-deaza-2′-deoxy-guanosine monophosphate (**6**): Yield was 27% for two steps. ^1^H-NMR (500 MH_Z_, D_2_O) δ (ppm) 6.54–6.51 (m, 1H), 4.87–4.84 (m, 1H), 4.17–4.11 (m, 2H), 4.00–3.98 (m, 1H), 3.34–3.28 (m, 1H), 2.36–2.31 (m, 1H). ^31^P-NMR (202 MHz, D_2_O) δ (ppm) 1.68. HR-ESI-MS (*m*/*z*) calcd. for C_11_H_12_N_4_O_7_Br_2_P [M − H]^−^: 500.8864, 502.8795, 504.8775, found: 500.8864, 502.8831, 504.8819.

7,8-di-Iodo-7-deaza-2′-deoxy-guanosine monophosphate (**7**): Yield was 9% for two steps. ^1^H-NMR (500 MH_Z_, D_2_O) δ (ppm) 6.48 (t, 1H, *J* = 8.4 Hz), 4.87–4.84 (m, 1H), 4.17–4.09 (m, 2H), 3.99–3.94 (m, 1H), 3.36–3.30 (m, 1H), 2.34–2.28 (m, 1H). ^31^P-NMR (202 MHz, D_2_O) δ (ppm) 3.98. HR-ESI-MS (*m*/*z*) calcd. for C_11_H_12_N_4_O_7_I_2_P [M − H]^−^: 596.8538, found: 596.8579.

8-Iodo-7-deaza-2′-deoxy-guanosine monophosphate (**8**): Yield was 30% for two steps. ^1^H-NMR (500 MH_Z_, D_2_O) δ (ppm) 7.05 (s, 1H), 6.28 (t, 1H, *J* = 7.0 Hz), 4.88–4.85 (m, 1H), 4.22–4.16 (m, 1H), 4.15–4.13 (m, 1H), 4.01–3.97 (m, 1H), 3.41–3.35 (m, 1H), 2.38–2.33 (m, 1H). ^31^P-NMR (202 MH_Z_, D_2_O) δ (ppm) 3.42. HR-ESI-MS (*m*/*z*) calcd. for C_11_H_13_N_4_O_7_IP [M − H]^−^: 470.9572, found: 470.9591.

#### 3.1.4. General Procedure for the Synthesis of 5′-Triphosphate Compounds

The solution of 2-chloro-4-*H*-1,3,2-benzodioxaphosphorin-4-one (0.26 mmol) in DMF (0.35 mL) and tributyl amine (0.5 mL) were added dropwise to the solution of tributyl ammonium pyrophosphate (0.26 mmol) in DMF (0.35 mL) and stirred for 30 min under an argon atmosphere at room temperature. This reaction mixture was added to the solution of the corresponding diol compounds (**1**, **2**, and **3**) (0.13 mmol) in DMF (0.1 mL). After stirring for 2 h, the oxidation reagent (4 mL, 1.0% I_2_ in pyridine/H_2_O = 98/2) was added to the reaction mixture and stirred for 10 min, and then the reaction was quenched by 5.0% NaHSO_3_ solution (4 mL). After ethanol precipitation, the resulting residue was purified by reversed-phase HPLC to give corresponding monophosphate compounds as a white material after lyophilization. (HPLC conditions: A: 0.1 M TEAA, B: MeCN; Flow rate: 1.0 mL/min; Shiseido MG1 (4.6 × 250 mm); UV detector: 254 nm; 35 °C).

7,8-di-Chloro-7-deaza-2′-deoxy-guanosine triphosphate (**9**): Yield was 30%. ^1^H-NMR (500 MH_Z_, D_2_O) δ (ppm) 6.50 (t, 1H, *J* = 7.5 Hz), 4.87–4.84 (m, 1H), 4.33–4.31 (m, 1H), 4.22–4.17 (m, 2H), 3.32–3.28 (m, 1H), 2.36–2.31 (m, 1H). ^31^P-NMR (202 MHz, D_2_O) δ (ppm) −10.9, −11.3, −23.3. HR-ESI-MS (*m*/*z*) calcd. for C_11_H_14_N_4_O_13_Cl_2_P_3_ [M − H]^−^: 572.9195, 574.9123, 576.9093, found: 572.9149, 574.9122, 576.9090.

7,8-di-Bromo-7-deaza-2′-deoxy-guanosine triphosphate (**10**): Yield was 14%. ^1^H-NMR (500 MH_Z_, D_2_O) δ (ppm) 6.52 (t, 1H, *J* = 7.5 Hz), 4.92–4.90 (m, 1H), 4.39–4.37 (m, 1H), 4.23–4.17 (m, 2H), 3.38–3.31 (m, 1H), 2.35–2.30 (m, 1H). ^31^P-NMR (202 MHz, D_2_O) δ (ppm) −9.4, −11.1, −23.0. HR-ESI-MS (*m*/*z*) calcd. for C_11_H_14_N_4_O_13_Br_2_P_3_ [M − H]^−^: 660.8142, 662.8122, 664.8102, found: 660.8171, 662.8152, 664.8132.

7,8-di-Iodo-7-deaza-2′-deoxy-guanosine triphosphate (**11**): Yield was 14%. ^1^H-NMR (500 MH_Z_, D_2_O) δ (ppm) 6.48 (t, 1H, *J* = 7.5 Hz), 4.94–4.92 (m, 1H), 4.40–4.36 (m, 1H), 4.24–4.15 (m, 2H), 3.38–3.30 (m, 1H), 2.31–2.26 (m, 1H). ^31^P-NMR (202 MHz, D_2_O) δ (ppm) −9.9, −11.1, −23.0 HR-ESI-MS (*m*/*z*) calcd. for C_11_H_14_N_4_O_13_I_2_P_3_ [M − H]^−^: 756.7865, found: 756.7902.

### 3.2. Hydrolysis of 7,8-Dihalogenated-7-deaza-dGTP, 8-CF_3_-7-deaza-dGTP, and 8-oxo-dGTP by MTH1

The reaction mixture was pre-incubated at 37 °C for 10 min before the addition of MTH1 to a final volume of 100 L (final concentration: 20 mM Tris-HCl, pH 7.5, 4 mM MgCl_2_, 40 mM NaCl, 80 g/mL bovine serum albumin (BSA), 8 mM dithiothreitol (DTT), 10% glycerol; 0.5 pmol MTH1; 50 μM 7,8-dihalogenated-7-deaza-dGTP (**7**, **10**, and **13**), 8-CF_3_-7-deaza-dGTP (**4**), and 8-oxo-dGTP). The resulting solutions were incubated at 37 °C for various times and quenched with 100 μL of stop solution (5 mM EDTA). All samples were loaded on ice before the analysis with HPLC (HPLC conditions: A: 0.1 M TEAA, B: CH_3_CN; Flow rate: 1.0 mL/min; Shiseido MG1 (4.6 × 250 mm); UV detector: 254 nm; column oven: 35 °C).

### 3.3. Inhibitory Effects of New 7-Deaza-dG Derivatives on the Hydrolytic Activity of MTH1

Twenty microliters of MTH1 buffer (100 mM Tris-HCl, pH 7.5, 20 mM MgCl_2_, 200 mM NaCl, 400 g/mL BSA, 40 mM DTT, and 50% glycerol), 10 μL (500 μM) of 8-oxo-dGTP, 55 μL of MilliQ, and 10 μL of various concentrations of substituted 7-deaza-dG derivatives were pre-incubated at 37 °C for 10 min (concentrations used: 7-deaza-dG, 7,8-dihalogenated-7-deaza-dG were 0, 0.5, 1, 2.5, 5, 10, 25, 50, 100, and 250 μM in 1% DMSO; 8-CF_3_-7-deazadGMP (**3**) was 0, 0.05, 0.1, 0.25, 0.5, 1, 2.5, 5, 10, and 25 μM; 7,8-dihalogenated-7-deaza-dGMP and –dGTP were 0, 0.0125, 0.025, 0.05, 0.1, 0.25, 0.5, 1, and 2.5 μM). Five microliters of MTH1 (0.1 pmol/μL in MTH1 buffer) was added, and the reaction mixture was incubated at 37 °C for 10 min. The resulting solutions were quenched with 100 μL of stop solution (5 mM EDTA). All samples were loaded on ice before the analysis with HPLC (HPLC conditions: A: 0.1 M TEAA, B: MeCN; Flow rate: 1.0 mL/min; Shiseido MG1 (4.6 × 250 mm); UV detector: 254 nm; 35 °C). The relative hydrolysis ratio values (ratio with inhibitor/ratio without inhibitor) were plotted at each inhibitor concentration. IC_50_ values were assessed using images.

### 3.4. Molecular Docking

The three-dimensional (3D) crystal structure of MTH1 complexed with 8-oxo-dGTP (PDB ID: 5FSI) was downloaded from the Protein Data Bank (PDB). AutoDockTools 1.5.6 (ADT, http://autodock.scripps.edu/) was used to remove the water and oxo-dGTP molecules, add hydrogen atoms, merge the non-polar hydrogen atoms, check and repair missing atoms, and assign Gasteiger charges and the AD4 atom type for the 5FSI crystal structure. The area of the protein involved in the docking calculation was also set with a ADT of 60 × 60 × 60 Å^3^ grid size and the center x, y, and z coordinates were set at 14.1, 19.6, and 9.2, respectively, to cover the whole protein (blind docking). The MTH1 structure prepared was then saved in the .pdbqt format. The structure of 8-oxo-dGTP was extracted from the 5FSI crystal structure and used as the standard in the docking study. ADT was then used to prepare the docking input file of the 8-oxo-dGTP structure, which included the detection of torsion angles, setting the number of torsions to the maximum, the assignment of Gasteiger charges, and conversion into the .pdbqt format for flexible ligand docking using AutoDock Vina. The processed oxodGTP structure was redocked back into its crystal structure using AutoDock Vina with an optimized exhaustiveness at 400, employing 5 different seedings of docking approaches with an output of 20 final conformations for each docking (total docking output conformations = 100). The root-mean-square deviation (RMSD) values between the resulting redocked conformations and the original conformation in the crystal structure of the ligand were then calculated using Discovery Studio 4.5 (DS) software. These redocked conformations with RMSD values less than 5 Å, when compared to the 8-oxo-dGTP moiety in the 5FSI crystal structure, were grouped and defined as “Percentage of 8-oxo-dGTP-like Binding”. A conformation with the lowest aforementioned RMSD value was selected as the best conformation for the binding interaction analysis through a 2D-interaction diagram generated by DS. The value of the lowest binding energy corresponding to the best conformation, which represents its binding affinity, was also extracted from the docking result. The best performing triphosphate derivatives in vitro, 8-dihalogenated-7-deaza-dGTPs (**7**, **10**, and **13**), were then selected and prepared by modifying 8-oxo-dGTP using DS for subsequent molecular docking to predict their binding properties.

## 4. Conclusions

We herein designed and successfully synthesized various 7-substituted and 7,8-dihalogenated 7-deaza-dG derivatives. These derivatives were designed as molecules that bind more strongly to the 8-oxo-dGTP repair enzyme, MTH1, based on the findings of our previous studies. We found that 7,8-dihalogenated 7-deaza-dGTPs (**7**, **10**, and **13**) were not hydrolyzed by MTH1. The triphosphate part was not hydrolyzed, which is consistent with our previous findings and is a very important property. 7,8-Dichloro-7-deaza-dG derivatives (**5**, **6**, and **7**), 7,8-dibromo-7-deaza-dG derivatives (**8**, **9**, and **10**), and 7,8-diiodo-7-deaza-dG derivatives (**11**, **12**, and **13**) exerted markedly stronger inhibitory effects on the hydrolytic activity of MTH1 than previously reported corresponding 8-iodo-7-deaza-dG derivatives (**14**, **15**, and **16**). The present results confirmed that the involvement of phosphate groups was also very important for binding to MHT1. This result was supported by the *K*_i_ value. Furthermore, the inhibition mode was competitive inhibition with the substrate, according to steady-state kinetic parameters. The binding mode was also predicted by the computational molecular docking simulation, and these 7,8-dihalogenated-7-deaza-dGTP derivatives were able to bind at an active site of MTH1 and showed similar binding shapes with the substrate, 8-oxo-dGTP. The present results are the first example of the development of novel nucleotide analogues for strong binding to nucleotide repair enzymes. In the future, these compounds will be modified with membrane-permeable molecules and developed into chemical tools for the inhibition of repair enzyme activity, selectivity, and elucidation of detailed enzyme functions or their distribution in cells.

## Figures and Tables

**Table 1:** IC_50_ values of diol, monophosphate, and triphosphate derivatives ^1^.

Entry	R_1_=	R_2_=	R_3_=	Compound	IC_50_ (μM)
1	Cl	Cl	H	**1**	22.5
2	Br	Br	H	**2**	10.7
3	I	I	H	**3**	17.0
4	I	H	H	**4** ^2^	44.8
5	Cl	Cl	PO_3_H_2_	**5**	0.13
6	Br	Br	PO_3_H_2_	**6**	0.26
7	I	I	PO_3_H_2_	**7**	0.13
8	I	H	PO_3_H_2_	**8**	0.84
9	Cl	Cl	P_3_O_9_H_4_	**9**	0.16
10	Br	Br	P_3_O_9_H_4_	**10**	0.11
11	I	I	P_3_O_9_H_4_	**11**	0.13
12	I	H	P_3_O_9_H_4_	**12** ^2^	0.42
13	-	-	-	**TH287** ^2^	0.01

^1^ Reaction conditions: 20 mM Tris-HCl pH 7.5, 4 mM MgCl_2_, 40 mM NaCl, 80 μg/mL BSA, 8 mM DTT, 10% glycerol; 50 μM 8-oxo-dGTP, 5 nM MTH1; various concentrations of substituted 7-deaza-dG derivatives. Data are means of three or more independent experiments. ^2^ Data from ref. 28, *ChemBioChem* 17, 566 (2016).

**Table 2:** Steady-state kinetic parameters for the MTH1 hydrolysis of 8-oxo-dGTP in the presence and absence of new 7-deaza-dG derivatives as inhibitors ^1^.

Entry	Inhibitor	*k*_cat_ (s^−1^)	*K*_M_ (μM)	*K*_i_ (nM)
1	without	10.6	17.6	-
2	7,8-dichloro-7-deaza-dGMP (**5**)	9.7	117.8	37.0
3	7,8- dichloro-7-deaza-dGTP (**9**)	9.3	142.9	28.1
4	7,8-dibromo-7-deaza-dGMP (**6**)	9.1	151.5	27.9
5	7,8-dibromo-7-deaza-dGTP (**10**)	9.4	126.6	24.2
6	7,8-diiodo-7-deaza-dGMP (**7**)	10.7	157.2	26.8
7	7,8-diiodo-7-deaza-dGTP (**11**)	11.9	133.4	30.4

^1^ Conditions: 20 mM Tris-HCl pH 7.5, 4 mM MgCl_2_, 40 mM NaCl, 80 μg/mL BSA, 8 mM DTT, 10% glycerol, 10–250 μM 8-oxo-dGTP, and 2 nM MTH1, in the absence or presence of new 7-deaza-dG derivatives, incubation at 37 °C for 4 or 10 min in the absence or presence of compounds.

## Data Availability

Not applicable.

## Supplementary Materials

Supplementary Materials, NMR, Mass spectrum and ADME prediction results, may be found at https://www.mdpi.com/1422-0067/22/3/1274/s1. Figure S1: Predicted ADME parameter of compound **9**, Figure S2: Predicted ADME parameter of compound **10**, Figure S3: Predicted ADME parameter of compound **11**, Figure S4: Predicted ADME parameter of compound **12**.

## Abbreviations

MTH1	human mutt homolog 1, also called NUDT1
7-deaza-dG	7-deaza-2′-deoxyguanosine
8-oxo-dGTP	8-oxo-2′-deoxyguanosine-5′-triphosphate
8-oxo-dGMP	8-oxo-2′-deoxyguanosine-5′-monophosphate
ROS	reactive oxygen species
IC_50_	half maximal inhibitory concentration
*K*_i_	inhibition constant
NCS	*N*-chlorosuccinimide
NBC	*N*-bromosuccinimide
NIS	*N*-iodosuccinimide
POCl_3_	phosphoryl chloride
TBSCl	*tert*-butylchlorodimethylsilane
DMAP	4-dimethylaminopyridine
Et_3_N-3HF	triethylamine trihydrofluoride
TEA	triethylamine
DCC	*N*,*N’*-dicyclohexylcarbodiimide
DMF	*N*,*N*-dimethylformamide
HPLC	high performance liquid chromatography
Tris-HCl	tris(hydroxymethyl)aminomethane hydrochloride
BSA	bovine serum albumin
DTT	dithiothreitol
*K*_M_	michaelis constant
*k*_cat_	turnover number
Asp	aspartic acid
Trp	tryptophan
Lys	lysine
