# Supplementary material for: Development of MTH1-Binding Nucleotide Analogs Based on 7,8-Dihalogenated 7-Deaza-dG Derivatives
Source: Int J Mol Sci. 2021 Jan 28;22(3):1274. doi: 10.3390/ijms22031274 (PMC7866122; doi:10.3390/ijms22031274)
Supplement: Supplementary file 1 [file ijms-22-01274-s001.pdf]

Supplementary Materials

Development of MTH1-binding nucleotide analogs based on 7,8-dihalogenated 7-deaza-dG derivatives

Hui Shi, Ren Ishikawa, Choon Han Heh, Shigeki Sasaki, and Yosuke Taniguchi

Contents:

1. ^{1}H-NMR and High-Resolution (HR) mass spectrum of diol compounds **1~3**. p2

2. ^{1}H-NMR and HR mass spectrum of 3'-OAc compounds **14~17**. p5

3. ^{1}H-, ^{32}P-NMR and HR mass spectrum of monophosphate compounds **5~8**. p8

4. ^{1}H-, ^{32}P-NMR and HR mass spectrum of triphosphate compounds **9~11**. p12

5. Predicted ADME parameter of compounds **9~12** in Figure S1~S4 using SwissADME (http://www.swissadme.ch/index.php).

   Figure S1. Predicted ADME parameter of compound **9**. p16

   Figure S2. Predicted ADME parameter of compound **10**. p16

   Figure S3. Predicted ADME parameter of compound **11**. p17

   Figure S4. Predicted ADME parameter of compound **12**. p17

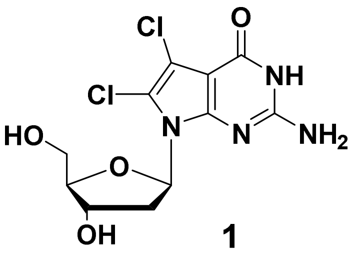
Cl
O
NH
Cl
HO
N
N
NH$_2$
O
OH

1

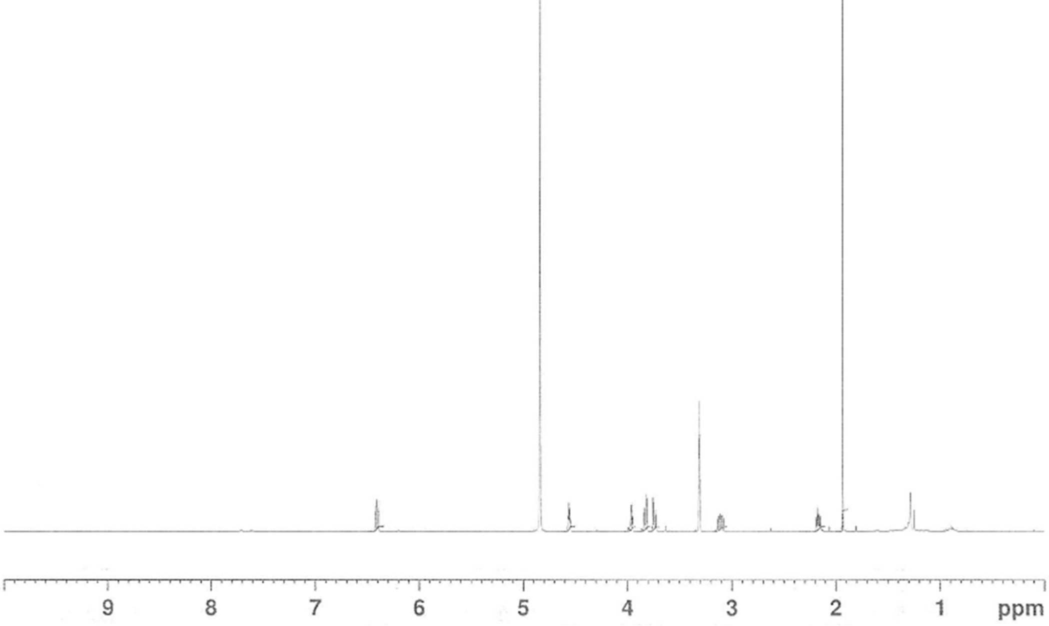
9
8
7
6
5
4
3
2
1
ppm

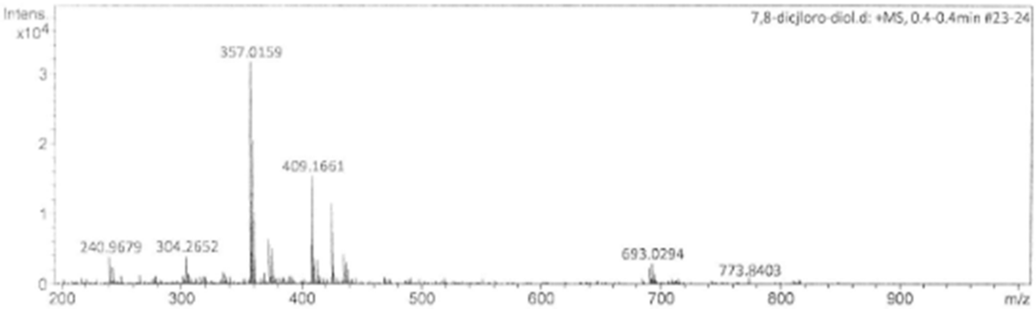
Intens.
x10^4
7,8-dicjloro-diol.d: +MS, 0.4-0.4min #23-24
357.0159
409.1661
240.9679
304.2652
693.0294
773.8403
m/z

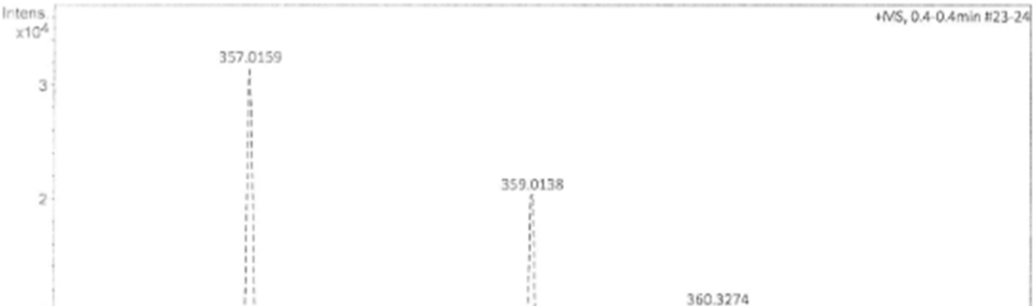
Intens.
x10^4
+MS, 0.4-0.4min #23-24
357.0159
359.0138
360.3274
358.0188
361.0130
360.0146
356.2635
357.2572
362.3238
m/z

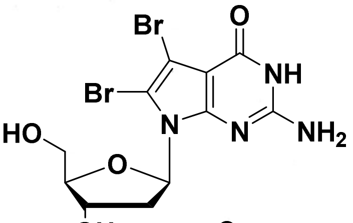
Br
O
NH
Br
HO
O
N
N
NH2
OH

2

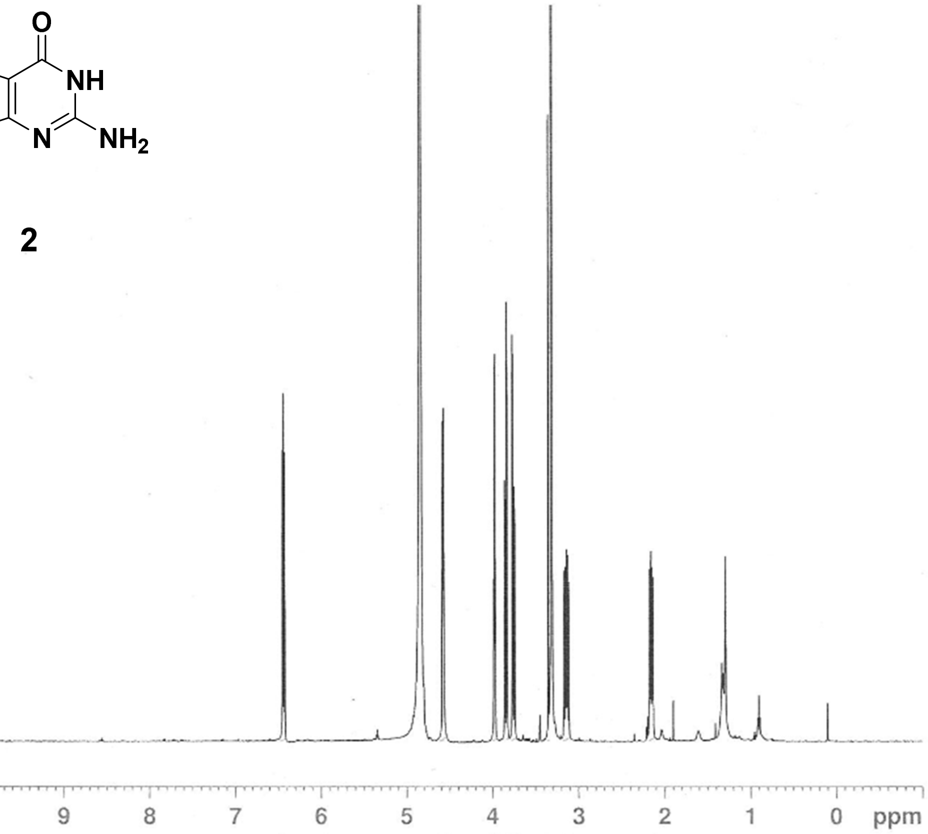
11 10 9 8 7 6 5 4 3 2 1 0 ppm

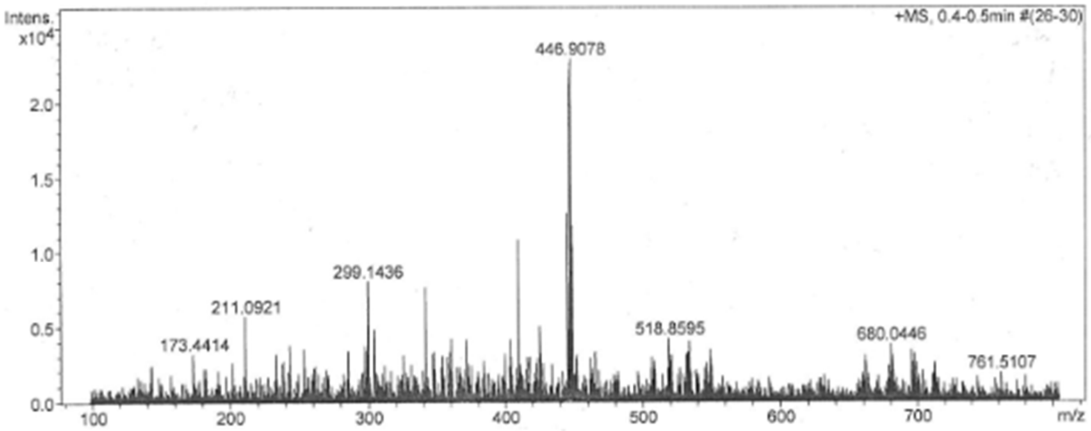
Intens.
x10^4
+MS, 0.4-0.5min #(26-30)
446.9078
299.1436
211.0921
173.4414
518.8595
680.0446
761.5107
2.0
1.5
1.0
0.5
0.0
100 200 300 400 500 600 700 m/z

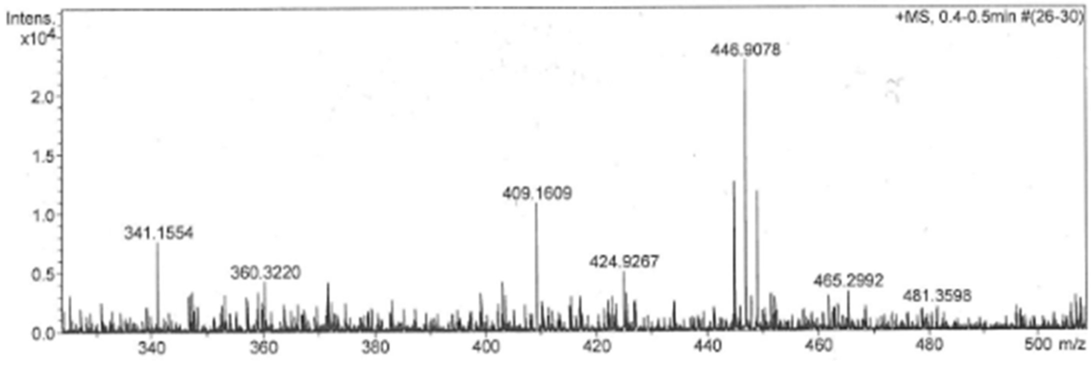
Intens.
x10^4
+MS, 0.4-0.5min #(26-30)
446.9078
409.1609
341.1554
360.3220
424.9267
465.2992
481.3598
2.0
1.5
1.0
0.5
0.0
340 360 380 400 420 440 460 480 500 m/z

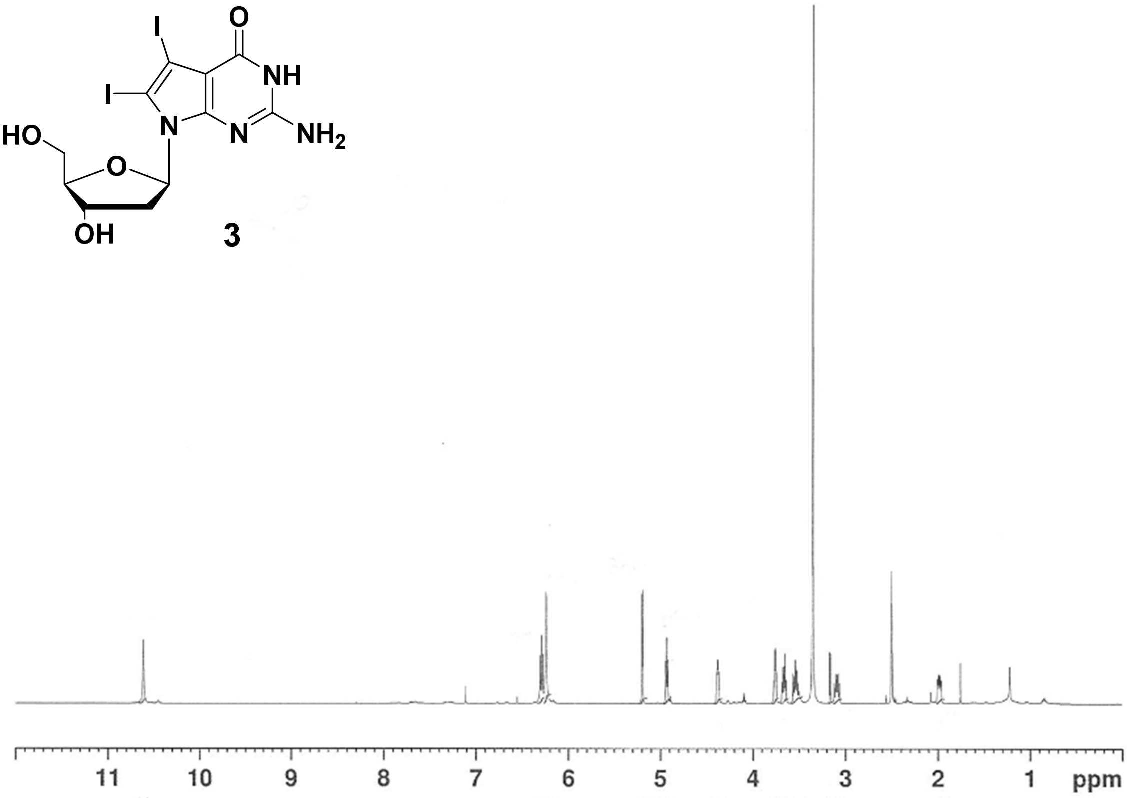
I
O
NH
I
HO
N
N
NH2
O
OH
3
11 10 9 8 7 6 5 4 3 2 1 ppm

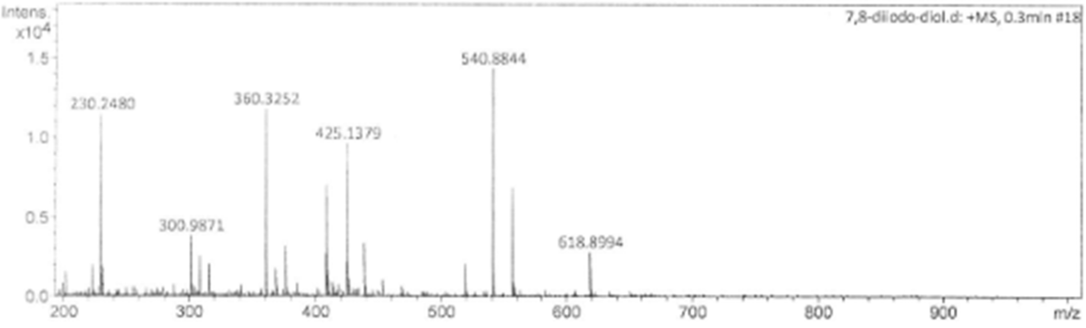
Intens.
x10^4
7,8-diiodo-diol.d: +MS, 0.3min #18
540.8844
230.2480
360.3252
425.1379
300.9871
618.8994
m/z

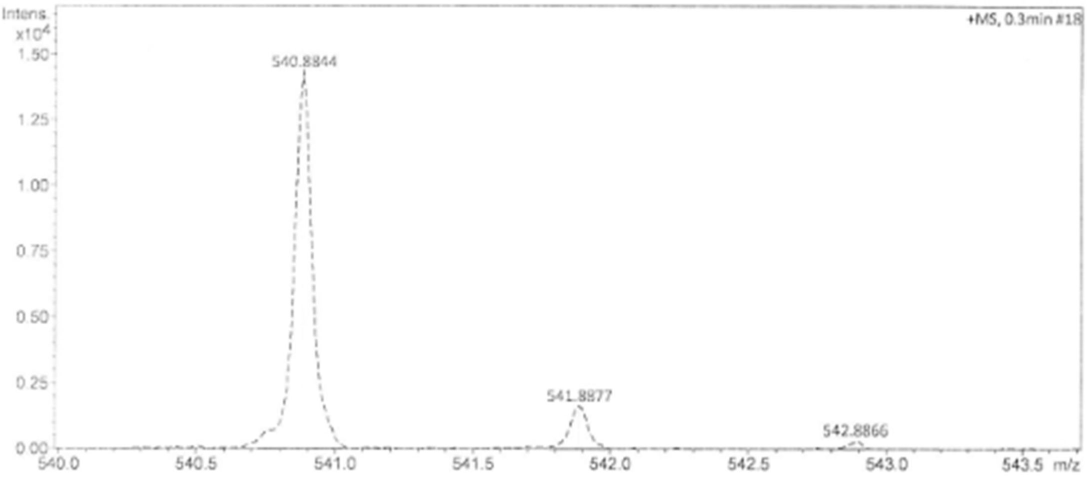
Intens.
x10^4
+MS, 0.3min #18
540.8844
541.8877
542.8866
m/z

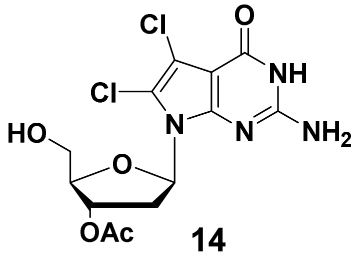

14

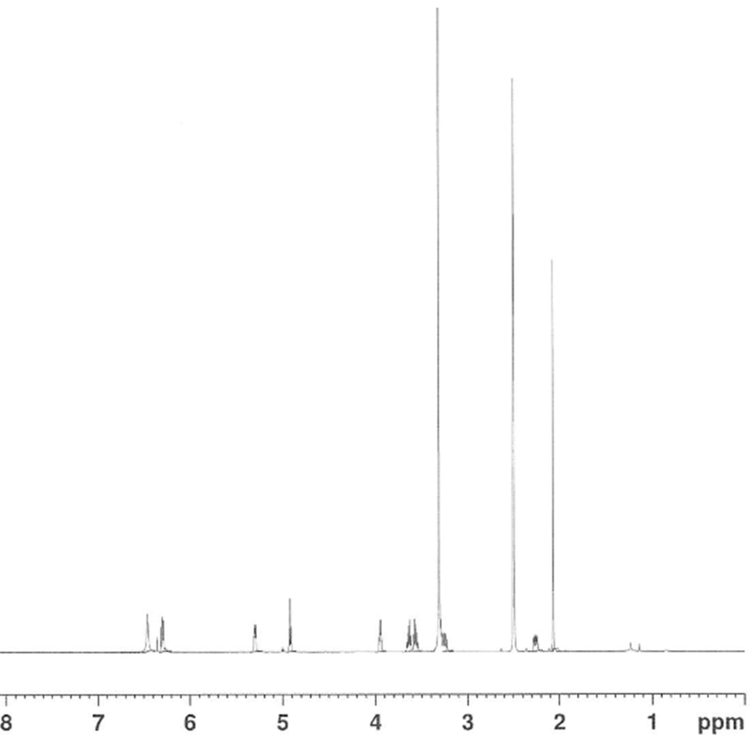

11
10
9
8
7
6
5
4
3
2
1
ppm

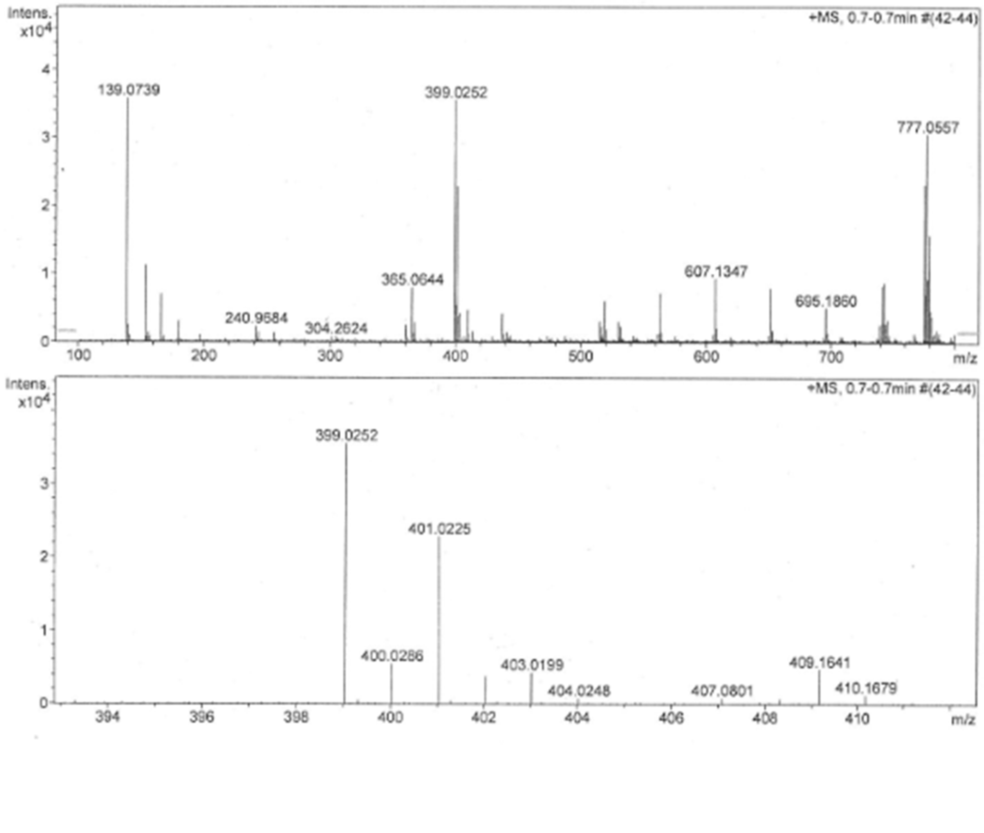

Intens.
x10⁴
+MS, 0.7-0.7min #(42-44)
139.0739
399.0252
777.0557
365.0644
607.1347
695.1860
240.9684
304.2624
m/z
Intens.
x10⁴
+MS, 0.7-0.7min #(42-44)
399.0252
401.0225
400.0286
403.0199
404.0248
407.0801
409.1641
410.1679
m/z

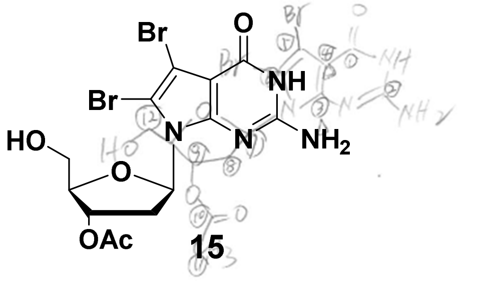

15

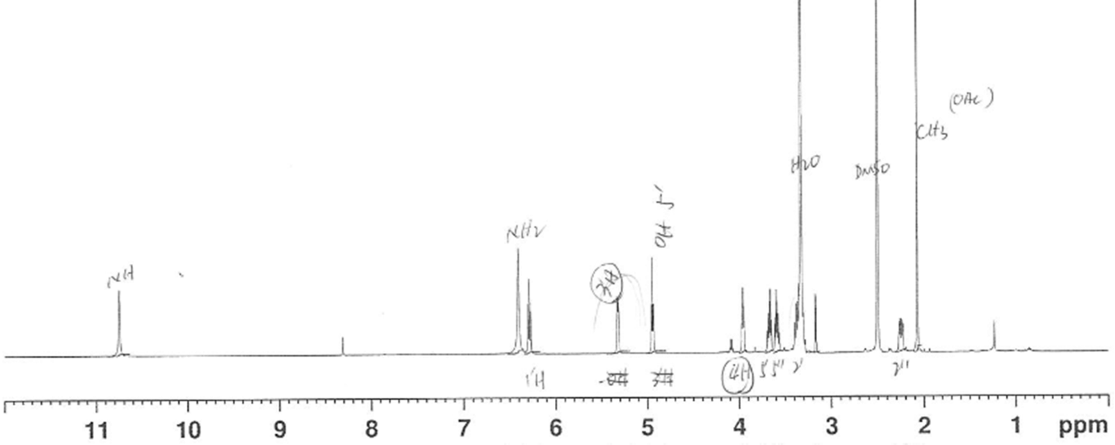

ppm
11
10
9
8
7
6
5
4
3
2
1

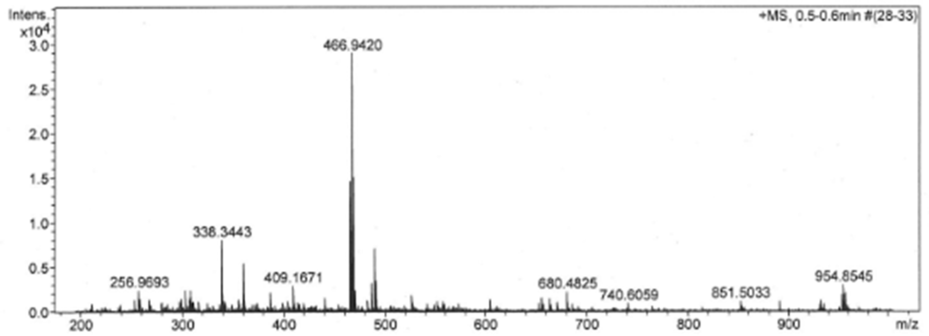

Intens.
x10^4
+MS, 0.5-0.6min #(28-33)
466.9420
338.3443
256.9693
409.1671
680.4825
740.6059
851.5033
954.8545
m/z

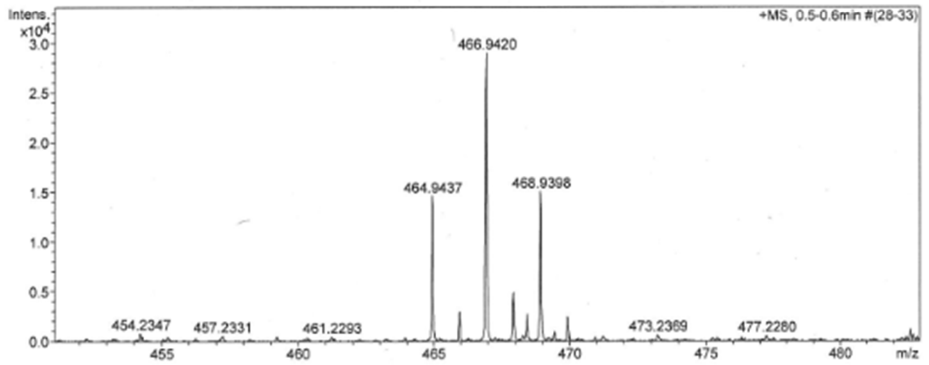

Intens.
x10^4
+MS, 0.5-0.6min #(28-33)
466.9420
464.9437
468.9398
454.2347
457.2331
461.2293
473.2369
477.2280
m/z

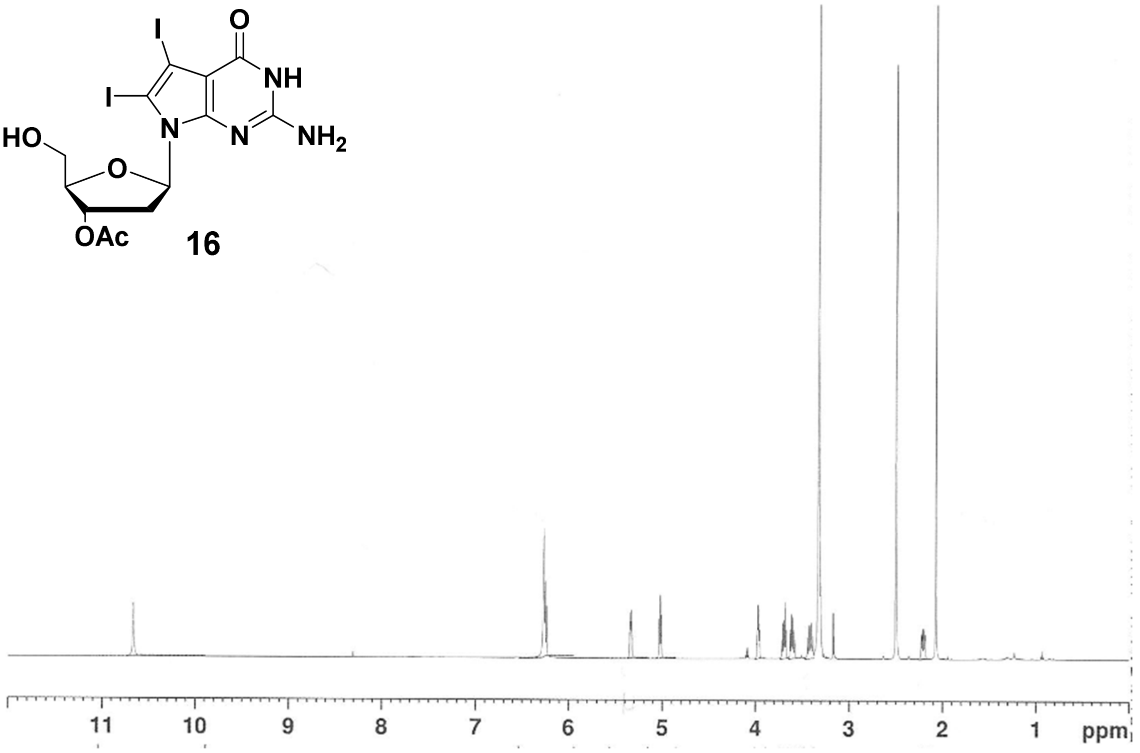

16
11 10 9 8 7 6 5 4 3 2 1 ppm

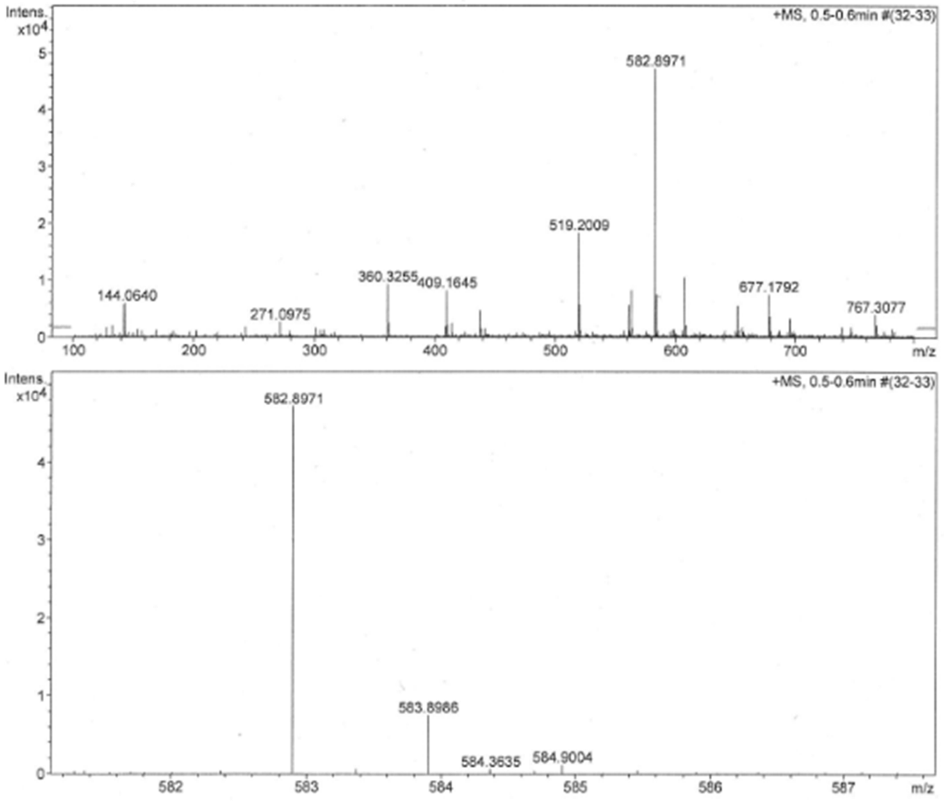

Intens.
x10^4
+MS, 0.5-0.6min #(32-33)
582.8971
519.2009
144.0640
271.0975
360.3255
409.1645
677.1792
767.3077
m/z
Intens.
x10^4
+MS, 0.5-0.6min #(32-33)
582.8971
583.8986
584.3635
584.9004
m/z

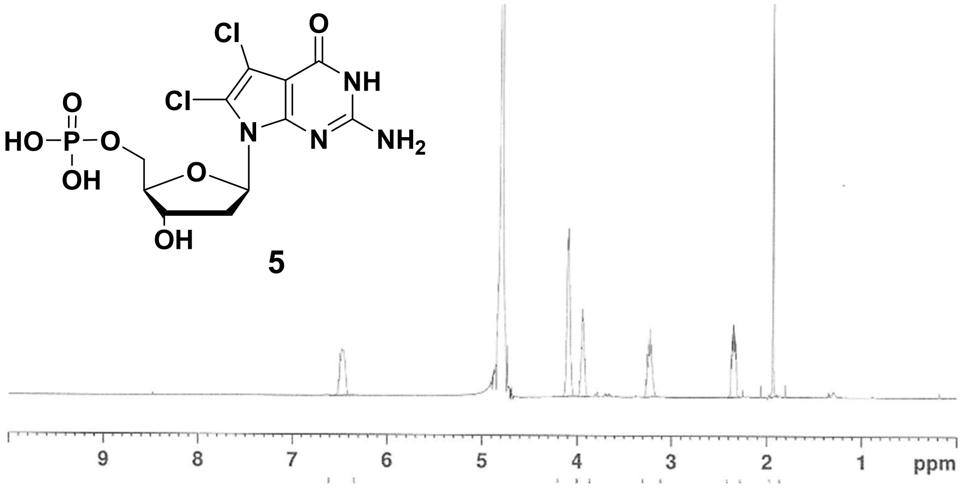
Cl
O
NH
Cl
HO
P
O
N
N
NH2
OH
O
OH
5
9
8
7
6
5
4
3
2
1
ppm

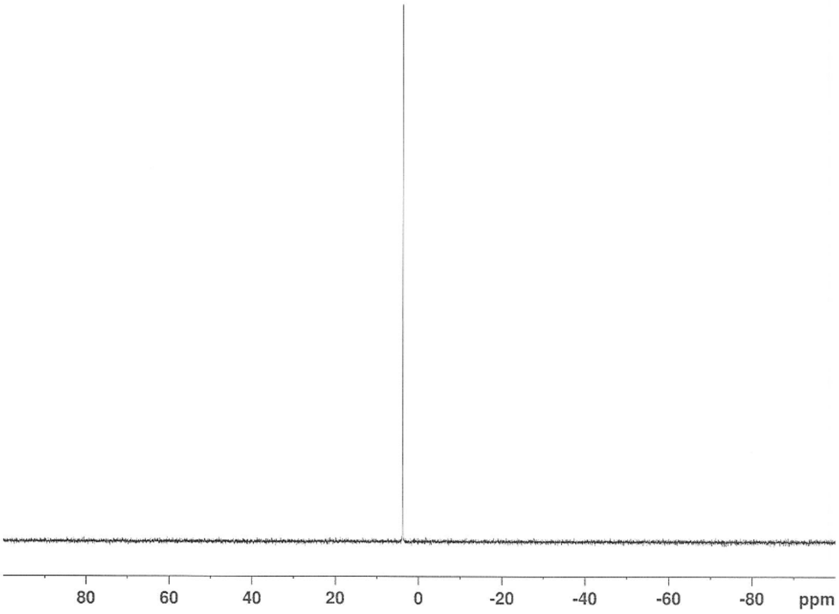
80
60
40
20
0
-20
-40
-60
-80
ppm

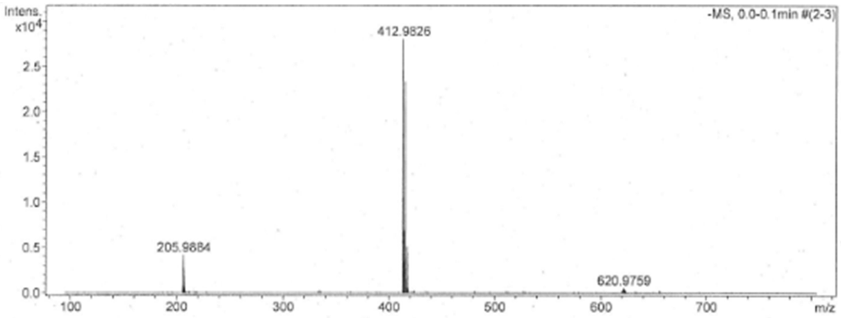
Intens.
x10^4
-MS, 0.0-0.1min #(2-3)
412.9826
205.9884
620.9759
2.5
2.0
1.5
1.0
0.5
0.0
100
200
300
400
500
600
700
m/z

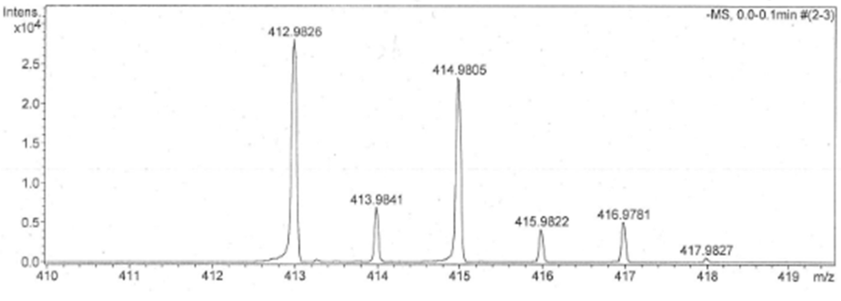
Intens.
x10^4
-MS, 0.0-0.1min #(2-3)
412.9826
414.9805
413.9841
415.9822
416.9781
417.9827
2.5
2.0
1.5
1.0
0.5
0.0
410
411
412
413
414
415
416
417
418
419
m/z

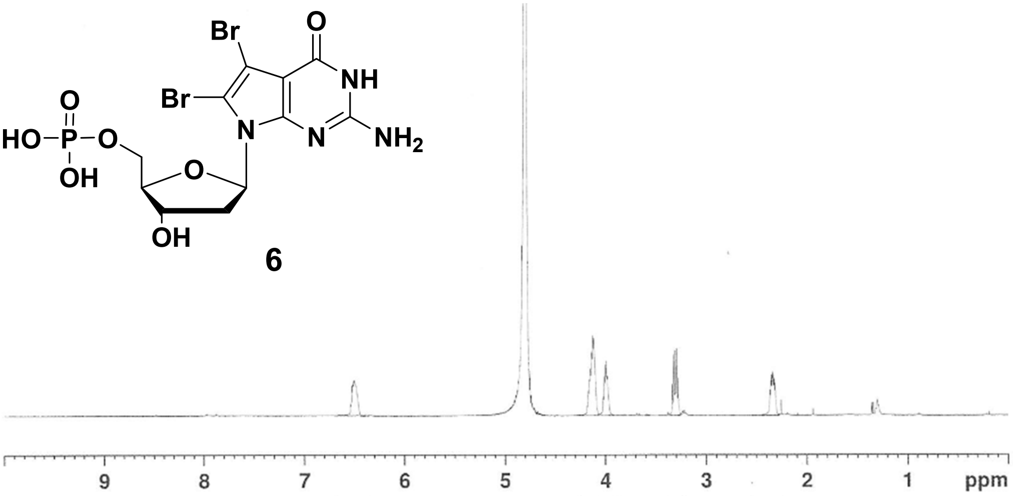
Br
Br
O
NH
N
N
NH2
HO
P
O
O
OH
O
OH
6
9
8
7
6
5
4
3
2
1
ppm

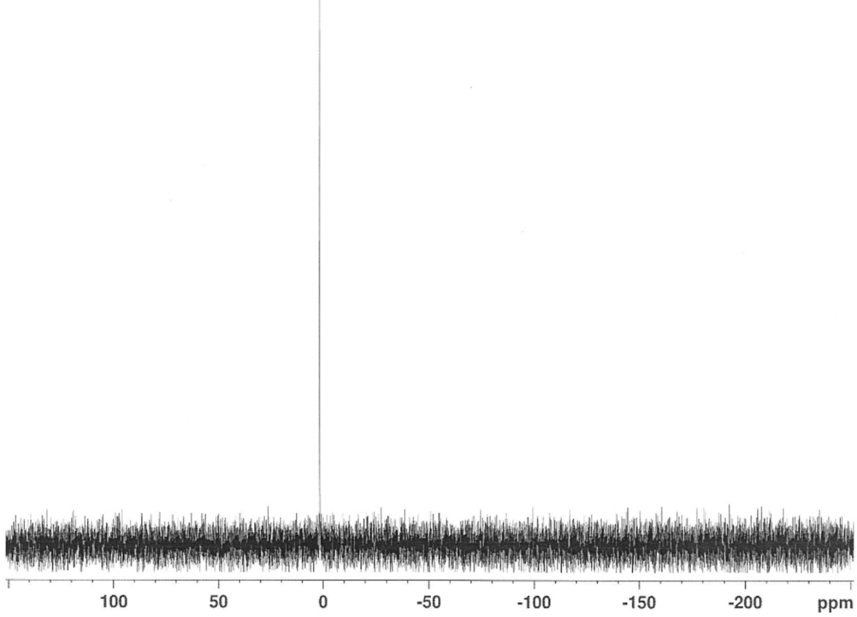
100
50
0
-50
-100
-150
-200
ppm

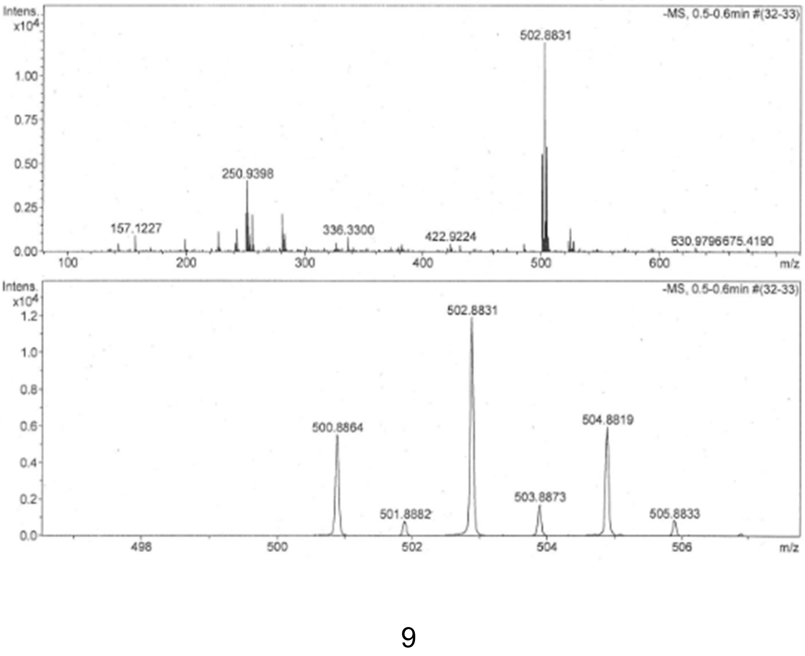
Intens.
x10^4
-MS, 0.5-0.6min #(32-33)
502.8831
250.9398
157.1227
336.3300
422.9224
630.9796675.4190
m/z
Intens.
x10^4
-MS, 0.5-0.6min #(32-33)
502.8831
500.8864
501.8882
503.8873
504.8819
505.8833
m/z

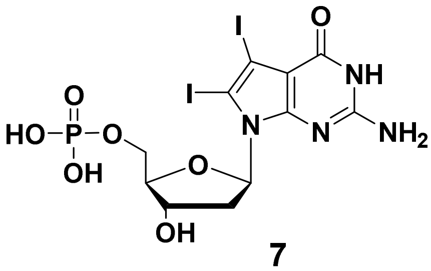

7

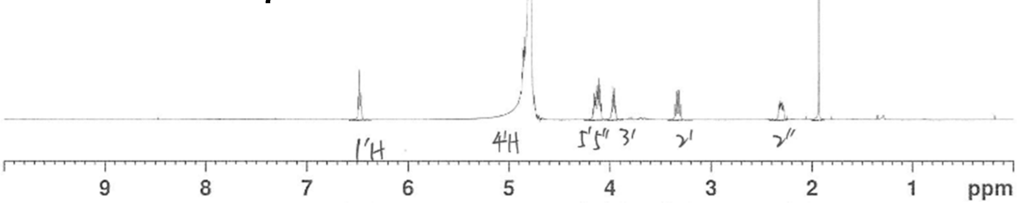
1'H
4'H
5'5" 3'
2'
2"
9
8
7
6
5
4
3
2
1
ppm

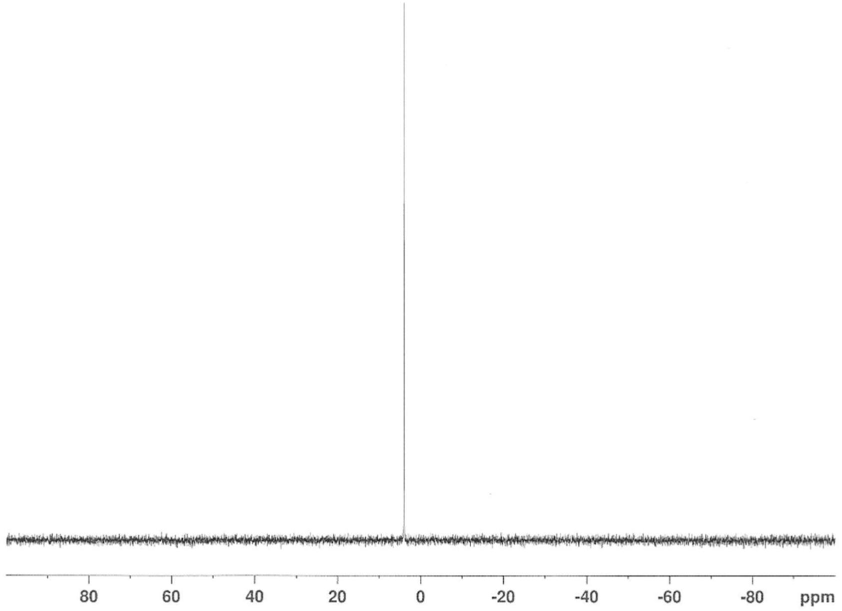
80
60
40
20
0
-20
-40
-60
-80
ppm

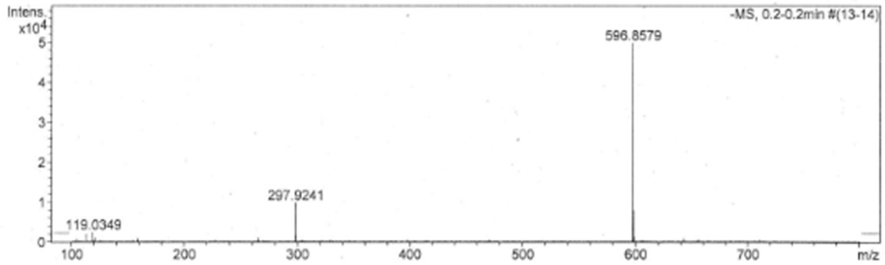
Intens.
x10^4
-MS, 0.2-0.2min #(13-14)
596.8579
297.9241
119.0349
100
200
300
400
500
600
700
m/z

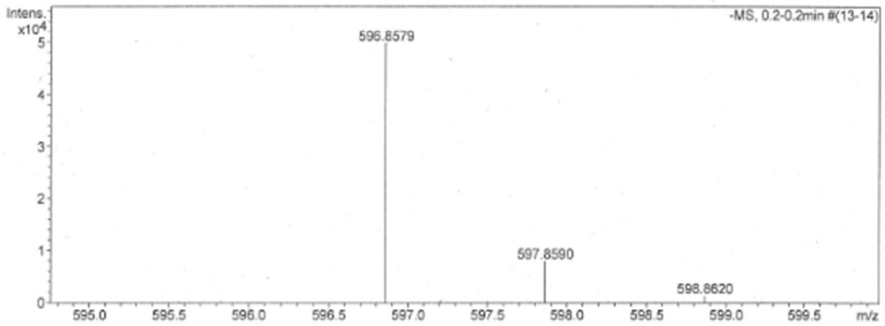
Intens.
x10^4
-MS, 0.2-0.2min #(13-14)
596.8579
597.8590
598.8620
595.0
595.5
596.0
596.5
597.0
597.5
598.0
598.5
599.0
599.5
m/z

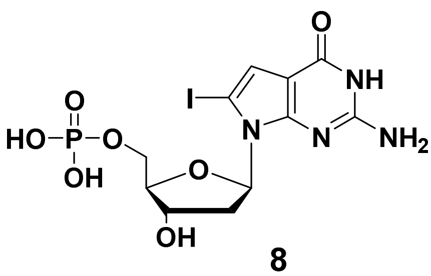

8

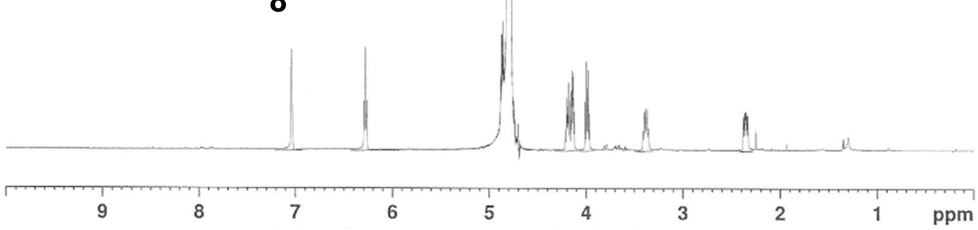

9
8
7
6
5
4
3
2
1
ppm

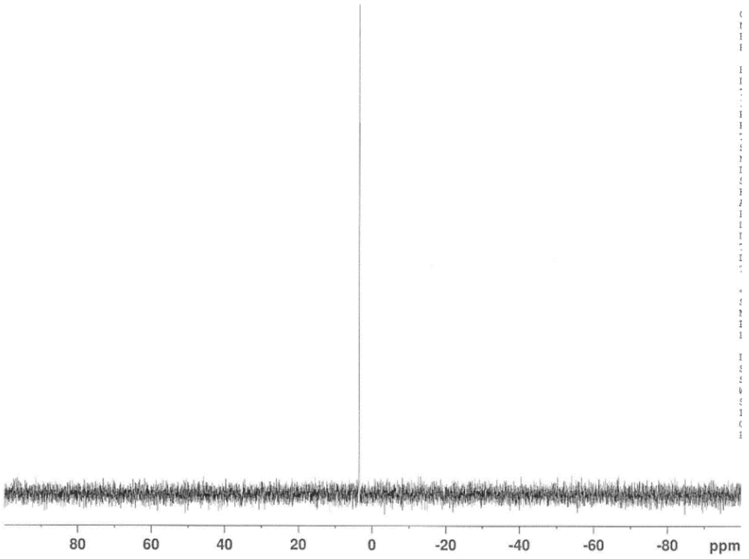

80
60
40
20
0
-20
-40
-60
-80
ppm

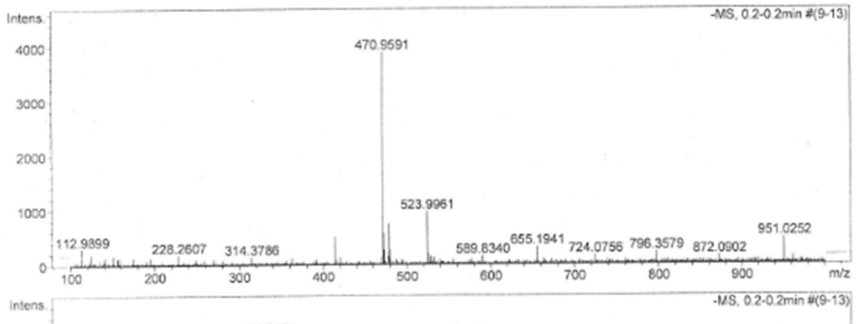

Intens.
-MS, 0.2-0.2min #(9-13)
470.9591
523.9961
112.9899
228.2607
314.3786
589.8340
655.1941
724.0756
796.3579
872.0902
951.0252
m/z

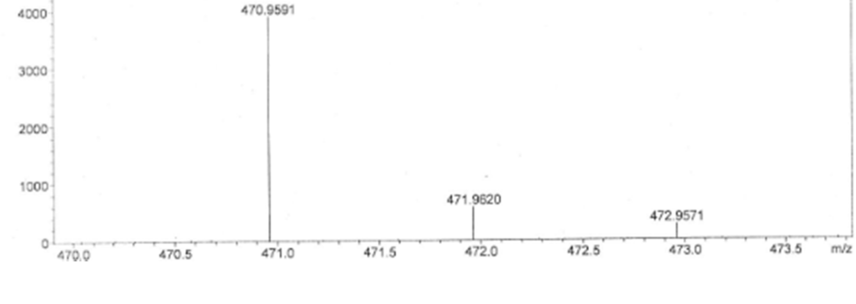

Intens.
-MS, 0.2-0.2min #(9-13)
470.9591
471.9620
472.9571
m/z

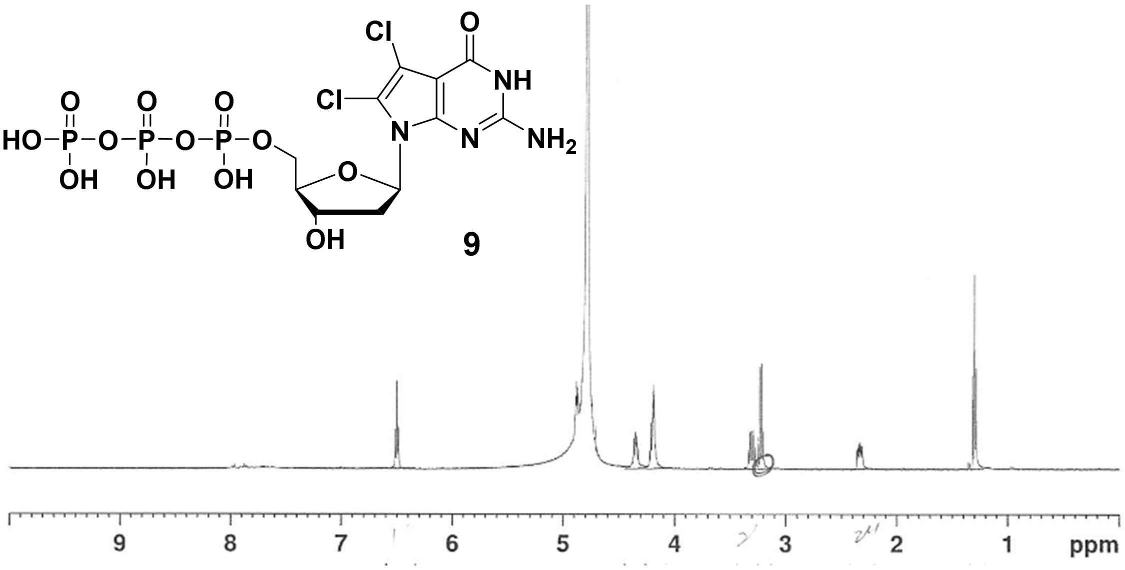
Cl
O
NH
Cl
N
N
NH2
HO
P
O
P
O
P
O
O
O
O
OH
OH
OH
O
OH
9
9
8
7
6
5
4
3
2
1
ppm

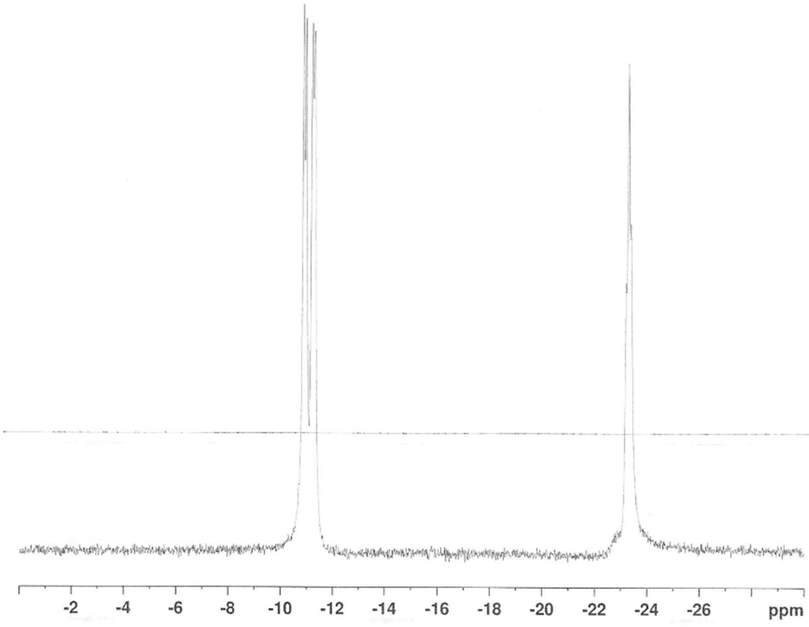
-2
-4
-6
-8
-10
-12
-14
-16
-18
-20
-22
-24
-26
ppm

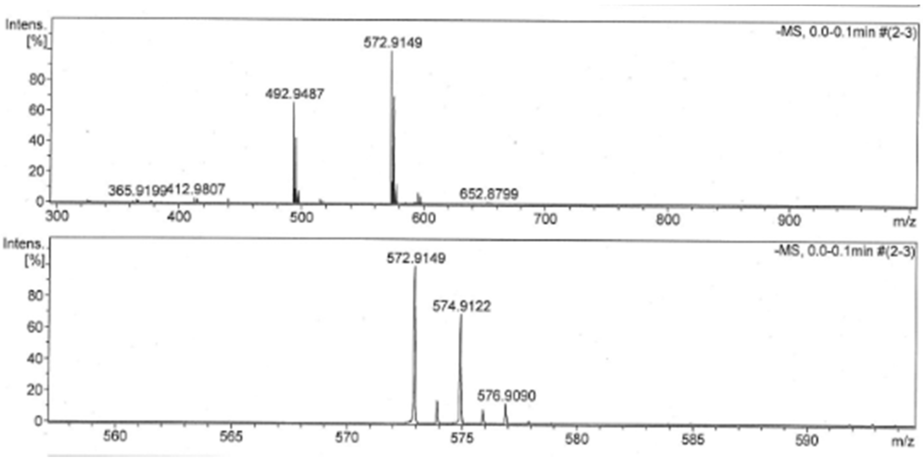
Intens.
[%]
-MS, 0.0-0.1min #(2-3)
572.9149
492.9487
365.9199
412.9807
652.8799
80
60
40
20
0
300
400
500
600
700
800
900
m/z
Intens.
[%]
-MS, 0.0-0.1min #(2-3)
572.9149
574.9122
576.9090
560
565
570
575
580
585
590
m/z

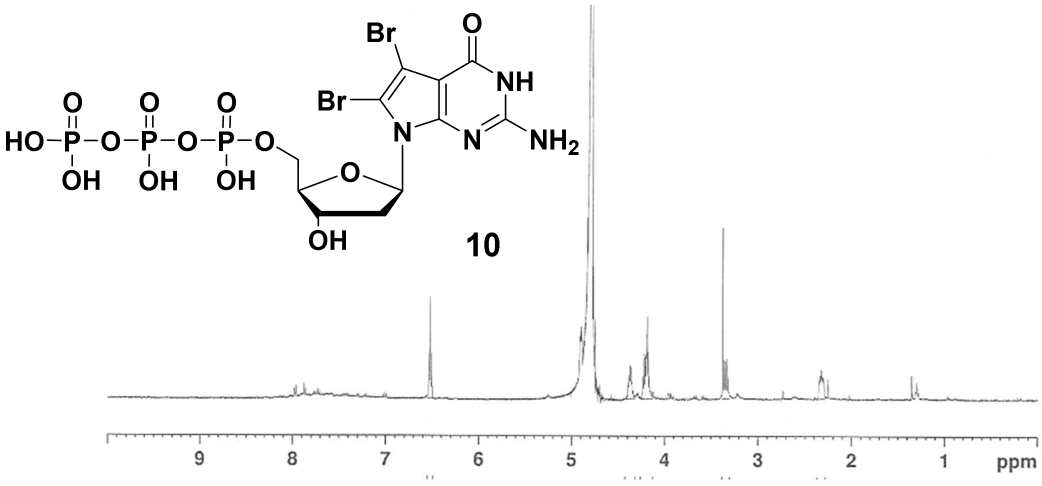
10
9 8 7 6 5 4 3 2 1 ppm

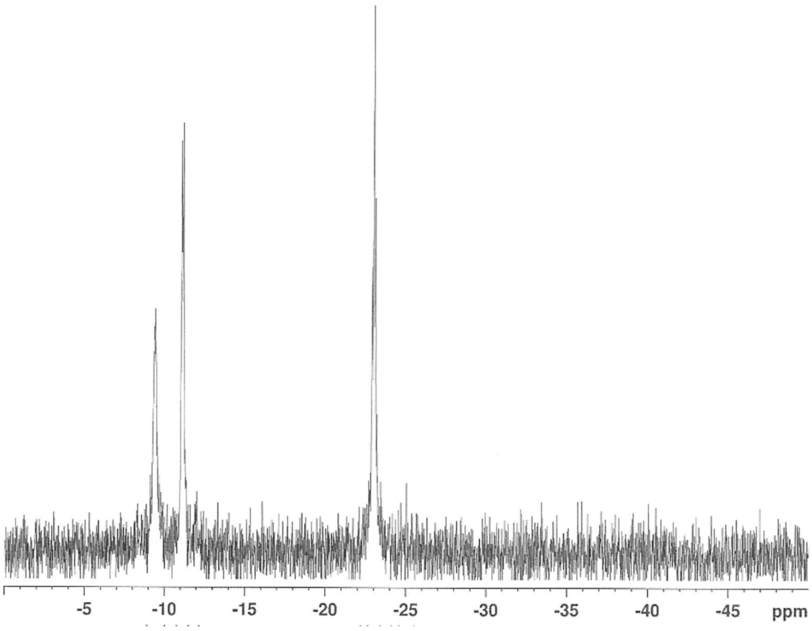
-5 -10 -15 -20 -25 -30 -35 -40 -45 ppm

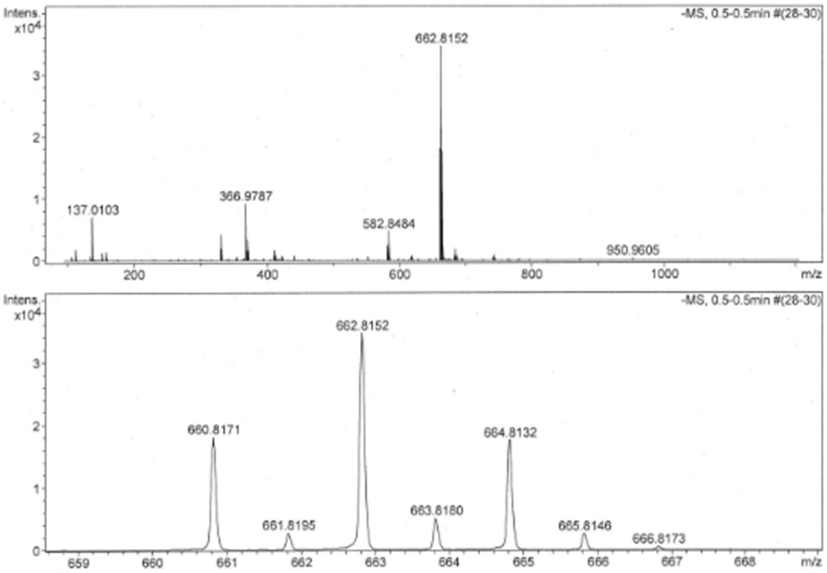
Intens. x10^4
-MS, 0.5-0.5min #(28-30)
662.8152
137.0103
366.9787
582.8484
950.9605
m/z
660.8171
661.8195
663.8180
664.8132
665.8146
666.8173

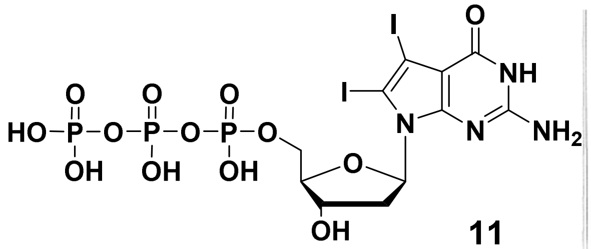

11

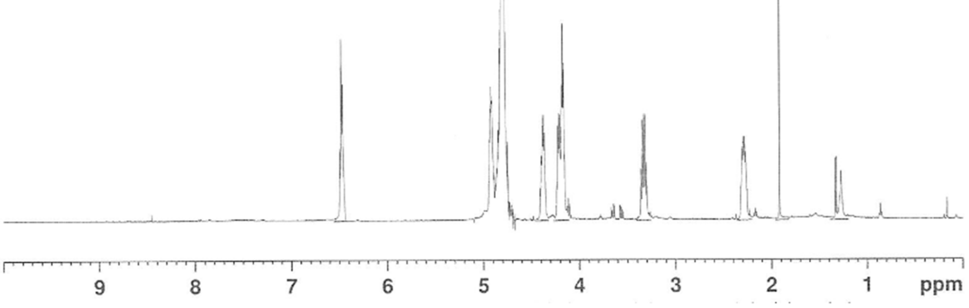
9 8 7 6 5 4 3 2 1 ppm

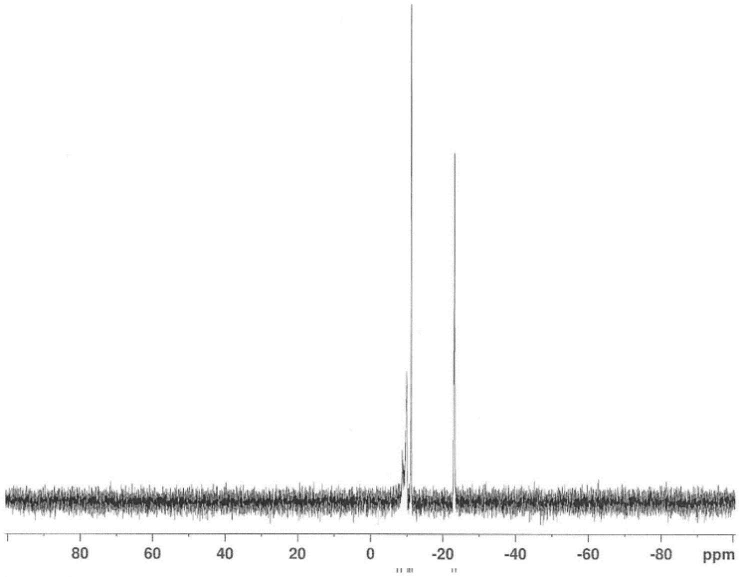
80 60 40 20 0 -20 -40 -60 -80 ppm

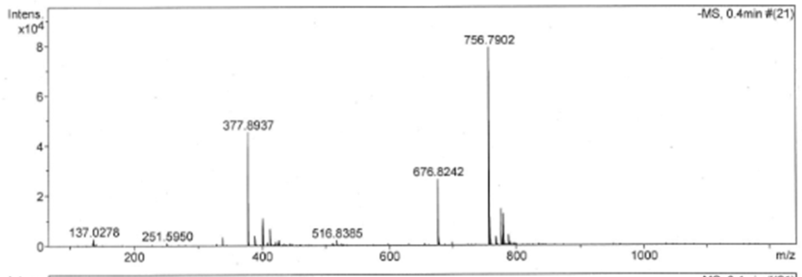
Intens. x10^4
-MS, 0.4min #(21)
756.7902
377.8937
676.8242
137.0278
251.5950
516.8385
m/z

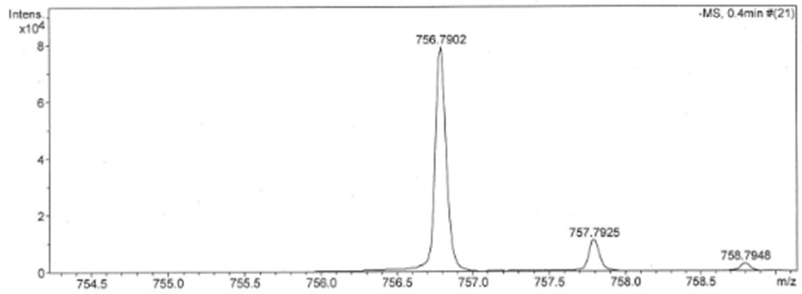
Intens. x10^4
-MS, 0.4min #(21)
756.7902
757.7925
758.7948
m/z

7,8-di-Chloro-7-deaza-2'-deoxy-guanosine triphosphate

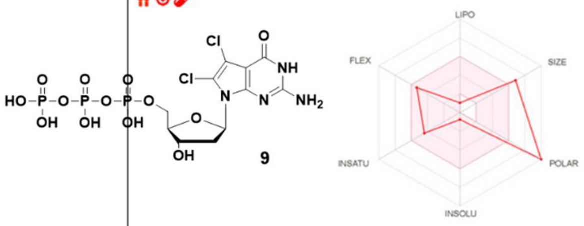

SMILES	O[C@H]1C[C@@H](O[C@@H]1COP(=O)(OP(=O)(OP(=O)(O)O)O)O)n1c(Cl)c(c2c1nc(N)[nH]c2=O)Cl

Physicochemical Properties	
Formula	C11H15Cl2N4O13P3
Molecular weight	575.08 g/mol
Num. heavy atoms	33
Num. arom. heavy atoms	9
Fraction Csp3	0.45
Num. rotatable bonds	8
Num. H-bond acceptors	14
Num. H-bond donors	7
Molar Refractivity	109.29
TPSA	295.41 Å²

Lipophilicity	
Log $P_{o/w}$ (iLOGP)	-0.48
Log $P_{o/w}$ (XLOGP3)	-3.69
Log $P_{o/w}$ (WLOGP)	0.29
Log $P_{o/w}$ (MLOGP)	-2.76
Log $P_{o/w}$ (SILICOS-IT)	-2.82
Consensus Log $P_{o/w}$	-1.89

Water Solubility	
Log *S* (ESOL)	-0.75
Solubility	1.01e+02 mg/ml ; 1.76e-01 mol/l
Class	Very soluble
Log *S* (Ali)	-1.93
Solubility	6.82e+00 mg/ml ; 1.19e-02 mol/l
Class	Very soluble
Log *S* (SILICOS-IT)	0.81
Solubility	3.73e+03 mg/ml ; 6.49e+00 mol/l
Class	Soluble

Pharmacokinetics	
GI absorption	Low
BBB permeant	No
P-gp substrate	Yes
CYP1A2 inhibitor	No
CYP2C19 inhibitor	No
CYP2C9 inhibitor	No
CYP2D6 inhibitor	No
CYP3A4 inhibitor	No
Log K_p (skin permeation)	-12.43 cm/s

Druglikeness	
Lipinski	No; 3 violations: MW>500, NorO>10, NHorOH>5
Ghose	No; 1 violation: MW>480
Veber	No; 1 violation: TPSA>140
Egan	No; 1 violation: TPSA>131.6
Muegge	No; 4 violations: XLOGP3<-2, TPSA>150, H-acc>10, H-don>5
Bioavailability Score	0.11

Medicinal Chemistry	
PAINS	0 alert
Brenk	1 alert: phosphor
Leadlikeness	No; 2 violations: MW>350, Rotors>7
Synthetic accessibility	4.62

Figure S1. Predicted ADME parameter of compound **9**.

7,8-di-Bromo-7-deaza-2'-deoxy-guanosine triphosphate

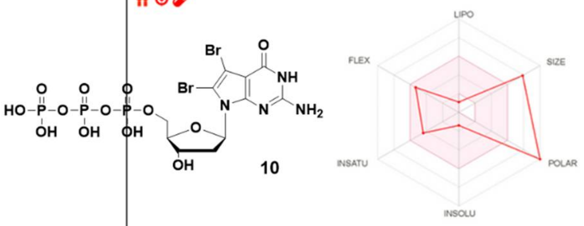

SMILES	O[C@H]1C[C@@H](O[C@@H]1COP(=O)(OP(=O)(OP(=O)(O)O)O)O)n1c(Br)c(c2c1nc(N)[nH]c2=O)Br

Physicochemical Properties	
Formula	C11H15Br2N4O13P3
Molecular weight	663.99 g/mol
Num. heavy atoms	33
Num. arom. heavy atoms	9
Fraction Csp3	0.45
Num. rotatable bonds	8
Num. H-bond acceptors	14
Num. H-bond donors	7
Molar Refractivity	114.67
TPSA	295.41 Å²

Lipophilicity	
Log $P_{o/w}$ (iLOGP)	-0.69
Log $P_{o/w}$ (XLOGP3)	-3.56
Log $P_{o/w}$ (WLOGP)	0.51
Log $P_{o/w}$ (MLOGP)	-2.50
Log $P_{o/w}$ (SILICOS-IT)	-2.75
Consensus Log $P_{o/w}$	-1.80

Water Solubility	
Log *S* (ESOL)	-1.39
Solubility	2.72e+01 mg/ml ; 4.10e-02 mol/l
Class	Very soluble
Log *S* (Ali)	-2.06
Solubility	5.78e+00 mg/ml ; 8.70e-03 mol/l
Class	Soluble
Log *S* (SILICOS-IT)	0.46
Solubility	1.92e+03 mg/ml ; 2.89e+00 mol/l
Class	Soluble

Pharmacokinetics	
GI absorption	Low
BBB permeant	No
P-gp substrate	Yes
CYP1A2 inhibitor	No
CYP2C19 inhibitor	No
CYP2C9 inhibitor	No
CYP2D6 inhibitor	No
CYP3A4 inhibitor	No
Log K_p (skin permeation)	-12.88 cm/s

Druglikeness	
Lipinski	No; 3 violations: MW>500, NorO>10, NHorOH>5
Ghose	No; 1 violation: MW>480
Veber	No; 1 violation: TPSA>140
Egan	No; 1 violation: TPSA>131.6
Muegge	No; 5 violations: MW>600, XLOGP3<-2, TPSA>150, H-acc>10, H-don>5
Bioavailability Score	0.11

Medicinal Chemistry	
PAINS	0 alert
Brenk	1 alert: phosphor
Leadlikeness	No; 2 violations: MW>350, Rotors>7
Synthetic accessibility	4.70

Figure S2. Predicted ADME parameter of compound **10**.

7,8-di-Iodo-7-deaza-2'-deoxy-guanosine triphosphate

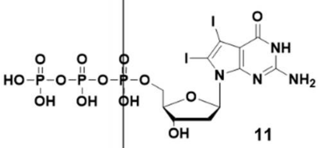

11

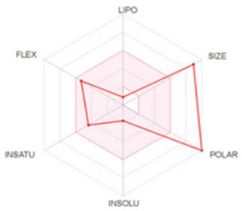

SMILES: O[C@H]1C[C@@H](O[C@@H]1COP(=O)(OP(=O)(OP(=O)(O)O)O)O)n1c(I)c(c2c1nc(N)[nH]c2=O)I

Physicochemical Properties	
Formula	C11H15I2N4O13P3
Molecular weight	757.99 g/mol
Num. heavy atoms	33
Num. arom. heavy atoms	9
Fraction Csp3	0.45
Num. rotatable bonds	8
Num. H-bond acceptors	14
Num. H-bond donors	7
Molar Refractivity	124.71
TPSA	295.41 Å²
**Lipophilicity**	
Log $P_{o/w}$ (iLOGP)	-0.29
Log $P_{o/w}$ (XLOGP3)	-3.94
Log $P_{o/w}$ (WLOGP)	0.19
Log $P_{o/w}$ (MLOGP)	-2.24
Log $P_{o/w}$ (SILICOS-IT)	-2.17
Consensus Log $P_{o/w}$	-1.69

Water Solubility	
Log *S* (ESOL)	-1.73
Solubility	1.41e+01 mg/ml ; 1.86e-02 mol/l
Class	Very soluble
Log *S* (Ali)	-1.67
Solubility	1.63e+01 mg/ml ; 2.16e-02 mol/l
Class	Very soluble
Log *S* (SILICOS-IT)	0.41
Solubility	1.93e+03 mg/ml ; 2.55e+00 mol/l
Class	Soluble
**Pharmacokinetics**	
GI absorption	Low
BBB permeant	No
P-gp substrate	Yes
CYP1A2 inhibitor	No
CYP2C19 inhibitor	No
CYP2C9 inhibitor	No
CYP2D6 inhibitor	No
CYP3A4 inhibitor	No
Log K_p (skin permeation)	-13.72 cm/s
**Druglikeness**	
Lipinski	No; 3 violations: MW>500, NorO>10, NHorOH>5
Ghose	No; 1 violation: MW>480
Veber	No; 1 violation: TPSA>140
Egan	No; 1 violation: TPSA>131.6
Muegge	No; 5 violations: MW>600, XLOGP3<-2, TPSA>150, H-acc>10, H-don>5
Bioavailability Score	0.11
**Medicinal Chemistry**	
PAINS	0 alert
Brenk	2 alerts: iodine, phosphor
Leadlikeness	No; 2 violations: MW>350, Rotors>7
Synthetic accessibility	4.89

Figure S3. Predicted ADME parameter of compound **11**.

8-Iodo-7-deaza-2'-deoxy-guanosine triphosphate

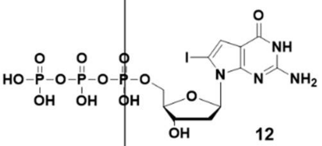

12

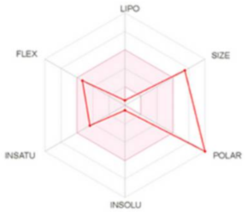

SMILES: O[C@H]1C[C@@H](O[C@@H]1COP(=O)(OP(=O)(OP(=O)(O)O)O)O)n1c(I)cc2c1nc(N)[nH]c2=O

Physicochemical Properties	
Formula	C11H16IN4O13P3
Molecular weight	632.09 g/mol
Num. heavy atoms	32
Num. arom. heavy atoms	9
Fraction Csp3	0.45
Num. rotatable bonds	8
Num. H-bond acceptors	14
Num. H-bond donors	7
Molar Refractivity	111.99
TPSA	295.41 Å²
**Lipophilicity**	
Log $P_{o/w}$ (iLOGP)	-1.99
Log $P_{o/w}$ (XLOGP3)	-4.59
Log $P_{o/w}$ (WLOGP)	-0.41
Log $P_{o/w}$ (MLOGP)	-3.03
Log $P_{o/w}$ (SILICOS-IT)	-3.18
Consensus Log $P_{o/w}$	-2.64

Water Solubility	
Log *S* (ESOL)	-0.55
Solubility	1.79e+02 mg/ml ; 2.84e-01 mol/l
Class	Very soluble
Log *S* (Ali)	-0.99
Solubility	6.44e+01 mg/ml ; 1.02e-01 mol/l
Class	Very soluble
Log *S* (SILICOS-IT)	1.17
Solubility	9.37e+03 mg/ml ; 1.48e+01 mol/l
Class	Soluble
**Pharmacokinetics**	
GI absorption	Low
BBB permeant	No
P-gp substrate	No
CYP1A2 inhibitor	No
CYP2C19 inhibitor	No
CYP2C9 inhibitor	No
CYP2D6 inhibitor	No
CYP3A4 inhibitor	No
Log K_p (skin permeation)	-13.41 cm/s
**Druglikeness**	
Lipinski	No; 3 violations: MW>500, NorO>10, NHorOH>5
Ghose	No; 2 violations: MW>480, WLOGP<-0.4
Veber	No; 1 violation: TPSA>140
Egan	No; 1 violation: TPSA>131.6
Muegge	No; 5 violations: MW>600, XLOGP3<-2, TPSA>150, H-acc>10, H-don>5
Bioavailability Score	0.11
**Medicinal Chemistry**	
PAINS	0 alert
Brenk	2 alerts: iodine, phosphor
Leadlikeness	No; 2 violations: MW>350, Rotors>7
Synthetic accessibility	4.80

Figure S4. Predicted ADME parameter of compound **12**.
